# Characterizing Influenza surveillance systems performance: application of a Bayesian hierarchical statistical model to Hong Kong surveillance data

**DOI:** 10.1186/1471-2458-14-850

**Published:** 2014-08-15

**Authors:** Ying Zhang, Ali Arab, Benjamin J Cowling, Michael A Stoto

**Affiliations:** Department of Health Systems Administration, School of Nursing and Health Studies, Georgetown University, Washington, DC USA; Department of Mathematics and Statistics, Georgetown University, Washington, DC USA; School of Public Health, Li KaShing Faculty of Medicine, The University of Hong Kong, Hong Kong, Special Administrative Region, China

**Keywords:** Influenza surveillance, Internet-based surveillance, Biosurveillance, Bayesian hierarchical modeling, Information environment, Public awareness

## Abstract

**Background:**

Infectious disease surveillance is a process the product of which reflects both actual disease trends and public awareness of the disease. Decisions made by patients, health care providers, and public health professionals about seeking and providing health care and about reporting cases to health authorities are all influenced by the information environment, which changes constantly. Biases are therefore imbedded in surveillance systems; these biases need to be characterized to provide better situational awareness for decision-making purposes. Our goal is to develop a statistical framework to characterize influenza surveillance systems, particularly their correlation with the information environment.

**Methods:**

We identified Hong Kong influenza surveillance data systems covering healthcare providers, laboratories, daycare centers and residential care homes for the elderly. A Bayesian hierarchical statistical model was developed to examine the statistical relationships between the influenza surveillance data and the information environment represented by alerts from HealthMap and web queries from Google. Different models were fitted for non-pandemic and pandemic periods and model goodness-of-fit was assessed using common model selection procedures.

**Results:**

Some surveillance systems — especially *ad hoc* systems developed in response to the pandemic flu outbreak — are more correlated with the information environment than others. General practitioner (percentage of influenza-like-illness related patient visits among all patient visits) and laboratory (percentage of specimen tested positive) seem to proportionally reflect the actual disease trends and are less representative of the information environment. Surveillance systems using influenza-specific code for reporting tend to reflect biases of both healthcare seekers and providers.

**Conclusions:**

This study shows certain influenza surveillance systems are less correlated with the information environment than others, and therefore, might represent more reliable indicators of disease activity in future outbreaks. Although the patterns identified in this study might change in future outbreaks, the potential susceptibility of surveillance data is likely to persist in the future, and should be considered when interpreting surveillance data.

**Electronic supplementary material:**

The online version of this article (doi:10.1186/1471-2458-14-850) contains supplementary material, which is available to authorized users.

## Background

The threat of pandemic influenza has led to extensive efforts to strengthen the global influenza surveillance [1, 2], including the development of novel syndromic surveillance systems intended to identify potential outbreaks and track influenza in the population. Some focus on identifying influenza-like-illness (ILI) in clinical and other settings, while others search the Internet to identify disease outbreaks that might not have been recognized by the authorities [3, 4]. Having found high correlations with traditional surveillance systems and noting the benefits of timeliness and low cost, Internet-based surveillance systems have been widely recognized as important supplementary data sources for influenza surveillance [5].

Infectious disease surveillance is a process; the data available for analysis reflects not only disease status in the population (the signal) but also other non-random factors (the noise). Our research has shown that decisions made by patients, healthcare providers, and public health professionals about seeking and providing healthcare and about reporting cases to health authorities are all influenced by the information environment, which we define as the information the population is exposed to through media, the Internet, social networks, and so forth. And since the information environment changes constantly, surveillance data systems that depend on decisions by patients and health professionals are likely to be biased, possibly in different ways [6, 7]. Epidemiologists and public health practitioners typically recognize these potential biases qualitatively, and present their analysis of the available data with appropriate caveats. Public health practitioners and clinicians are also aware of the surge in medical resource utilization caused by the “worried well” in response to media coverage of disease outbreaks [8]. Awareness of these biases is often lost, however, at higher levels [9].

Some researchers assume that data on the information environment — such as Google searches and Twitter feeds — can be used as proxies that directly estimate disease transmission in the population as long as the “signal” can be separated from the “noise” (trends in the data reflecting public awareness rather than disease transmission *per se*) [3, 10, 11]. Others simply view information environment data as a direct proxy for disease transmission. Surveillance systems that are fast, inexpensive, decentralized, automated, and utilize the power of information technology seem to satisfy the need for a magic bullet in the digital era. Whether such systems work as expected, or are another example of “big data hubris” [12], remains an open question.

To address this issue in a rigorous way, the objectives of this study are to (1) develop a method to characterize the relationship between surveillance data and the information environment, (2) identify surveillance systems that more closely reflect actual disease trends rather than the information environment, therefore useful for tracking, and (3) understand the implications of the fact that some surveillance systems are more correlated with the information environment. In particular, we developed a Bayesian hierarchical statistical model that allows us to examine the relationship between surveillance data and information environment more formally than our previous analyses [6, 7]. This methodological paper is for public health surveillance specialists to better understand and improve the performance of data systems.

Our analysis uses influenza surveillance data and information environment proxy data (e.g. Google search and HealthMap) from Hong Kong during the pre-pandemic (2007-2008) and pandemic (June – November 2009) periods. Rather than thinking of influenza-related web queries and news being direct indicators of disease transmission in the population, we view them as indicators of the information environment. We built a Bayesian hierarchical statistical model to estimate the correspondence between individual surveillance data and the information environment proxy data. Although not employed in this analysis, the model has the potential to incorporate epidemiological expertise through informed prior distributions. The findings have enabled us to understand how each surveillance system is related to the information environment and disease status, which should eventually help public health practitioners interpret the influenza surveillance data for situational awareness purposes, as well as prioritizing resources to different surveillance systems given the specific decision-making needs.

## Methods

### Data description

#### Influenza surveillance data

The local public health agency in Hong Kong – Centre for Health Protection (CHP), conducts surveillance monitoring influenza-like-illness among a network of over 40 private general practitioners (GP) and 64 public sentinel general out-patient clinics (GOPC). The out-patient ILI surveillance network reports the proportion of outpatients with ILI [Fever (>38°C, oral or equivalent) **AND** Cough or sore throat] on weekly basis [13, 14]. During the Influenza A(H1N1)pdm09 virus infection (pH1N1) outbreak, eight designated flu clinics (DFC) were operated from June 13^th^ 2009 to May 23^rd^ 2010 [15], while the general out-patient clinics data were interrupted [16]. Hospital Authority, a public-sector organization that oversees all public hospitals, manages over 95% of the in-patient care [17]. The weekly numbers of hospitalization with principle diagnosis of pneumonia and influenza (P&I; ICD9 480‒487) and influenza (ICD9 487) were obtained from the Hospital Authority for age groups of 0-4,5-14,15-64 and ≥65 years. Respiratory specimens from sentinel out-patient clinics, general practitioners are routinely submitted to the Public Health Laboratory Centre (PHLC) of CHP for sentinel surveillance purpose, while specimens from other out-patient healthcare facilities and hospitals are routinely submitted to the PHLC for diagnostic purposes [13]. Weekly data on numbers of specimen received, numbers of specimen tested positive for influenza and proportion of specimen tested positive were obtained from PHLC for 1997-2009. Reliable estimates of weekly incidence rate of pH1N1 for age groups (5-14,15-19,20-29,30-39,40-49, 50-59) were constructed based on serological data and hospitalization data [18]. Summary of data sources are provided in Table 1.

**Table 1:** **Summary of Hong Kong Influenza surveillance data**

Y	Data	Description	Type	Period
**Y1**	**flu-HA**	Hospital admissions with principle diagnosis of influenza	Count	1998 to 2011
**Y2**	**GOPC**	Weekly ILI consultations at sentinel general out-patient clinics per 1,000 consultations	Percentage	1998 - 2011
**Y3**	**CCC/KG**	Percentage of children at the sentinel child care centers and kindergartens with fever	Percentage	2007 to 2011
**Y4**	**GP**	Weekly ILI consultations at sentinel physicians’ offices per 1,000 consultations	Percentage	1998 - 2011
**Y5**	**RHE**	Average number of elderly with fever per day per 1,000 elderly at the residential care homes	Percentage	2007 to 2011
**Y6**	**Lab(%pos)**	Percentage of confirmed influenza cases among all the tested samples	Percentage	1997 to 2009
**Y7**	**P&I-HA**	Hospital admissions with principle diagnosis of pneumonia and influenza	Count	1998 to 2011
**Y8**	**P&I-HA (0-15 yr)**	Hospital admissions with principle diagnosis of pneumonia and influenza for patient aged 0 to 15 years old.	Count	1998 to 2011
**Y9**	**P&I-HA (65+ yr)**	Hospital admissions with principle diagnosis of pneumonia and influenza for patient aged over 65 years old.	Count	1998 to 2011
**Y10**	**Lab(#spm)**	Number of specimens received for laboratory testing	Count	1997 to 2009
**Y11**	**Lab(#pos)**	Number of specimens tested positive for influenza virus	Count	1997 to 2009
**Pandemic Only Data Series**				
**Y12**	**DFC**	Number of ILI related patient visits at Designated Flu Clinics (8 clinics)	Count	June 13th 2009 – May 23rd 2010
**Y13**	**NID**	Number of confirmed influenza infection reports from all registered medical practitioners in HK	Count	April 27th 2009 – October 8th 2010
**Xc**	**Incidence rate (5-14 yr)**	Reliable estimate for incidence rate among children between 5 to 14 years old based on a serological study	Percentage	June 15th 2009 – November 22nd 2009
**Xa**	**Incidence rate (all age)**	Reliable estimate for incidence rate for all age group based on a serological study	Percentage	June 15th 2009 – November 22nd 2009

We used data from Hong Kong for this analysis because of the following reasons. First, Hong Kong has a population of high density and mobility. The heterogeneity in the disease transmission dynamics and the informational environment is less likely to be attributable to the geographic variability. Therefore, the influenza surveillance systems are presumably monitoring a relatively homogenous population under the same informational environment, which is an important presumption for this modeling approach. Also, in addition to the normal winter-spring peak, Hong Kong usually experiences a summer peak in July to August [19], which potentially doubles the data volume. Since 2004, the Hong Kong Centre for Health Protection (CHP) has been monitoring influenza activity using multiple surveillance systems summarized in a weekly surveillance dashboard. These include sentinel surveillance system based at Accidents and Emergency Department of public hospitals, private medical practitioners, general outpatient clinics, traditional Chinese medicine practitioners, childcare centres and kindergartens and residential care homes for the elderly, as well as more traditional laboratory and influenza-related hospitalization surveillance systems. Given the variety of data types available, as well as the availability of reliable estimate of incidence rate based on serological study during pandemic period as gold standard [18], Hong Kong is chosen for this project.

#### Information environment proxy data

Two data sources were identified as proxies for the information environment: HealthMap and web queries using Google search engine. HealthMap is an online information aggregator and surveillance platform that identifies, characterizes, and maps events of public health and medical importance in real-time [20]. For this project, the first author manually coded Chinese language feeds using data filtered by disease category and geographic location. All feeds in both English and Chinese categorized as respiratory illness and related to Hong Kong area were verified for the correctness and completeness based on the original sources. Among 4,695 feeds that were extracted from the earliest date available, 2,166 feeds posted between January 1^st^, 2007 and November 28^th^, 2009 were extracted and tabulated for this study. The inclusion and exclusion criteria for HealthMap alerts data can be found in Additional file 1: Table S1.

Given the bilingual cultural environment in Hong Kong, we developed an original search matrix covering the disease and behavior related indicators in both English and Chinese. The “seed” for keywords are first identified based on literature review [11]. Each seed keyword is then assessed for correlated search terms through the Google Correlates tool [21, 22]. Additional keywords are then identified through snowballing, until no more new keywords are shown in the correlation list. By using Google Insight for Search — now rebranded as Google Trends ― the search volume index is retrieved for each of the keyword individually for Hong Kong. The search volume index, also called the “interest over time” on the new Google Trends website, is the search volume of the individual search term divided by its maximum search volume during the user specified period [23] ― starting from 2004 to the latest available data.

**Figure 1:**
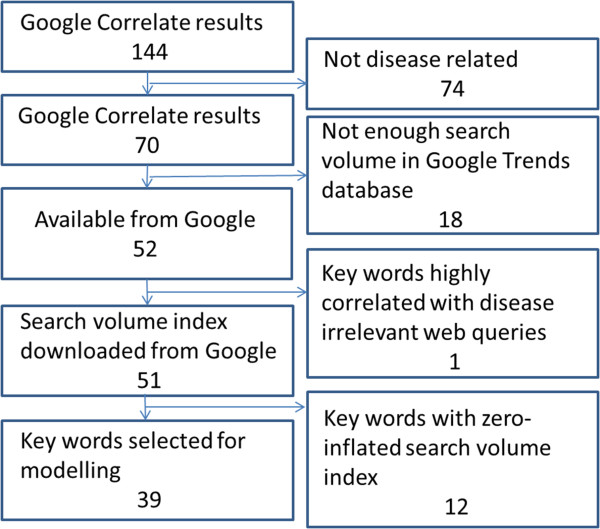
**Selection flowcharts for Google search keywords.**

Among the 144 original keywords through Google Correlate, 44 are in English and 100 are in Traditional Chinese, among which 74 are not disease related. 18 keywords have no search results from Google Trends due to the lack of search volume. Among the remaining 52 keywords, 20 are available on weekly basis and 32 are available on monthly basis. For keywords that only have monthly data, weekly estimates are estimated using the monthly value assuming the level stays the same through the month (Figure 1).

### Data preparation

All search index data are standardized. And for quality control purposes, the “Related terms” shown on the Google Trends website for each search keyword is also examined and documented, since some keywords are significantly correlated with non-disease related terms. In Additional file 1: Table S2, all keywords are listed under seven categories.

For influenza surveillance data, some researchers believe the flu activity in tropical areas like Hong Kong is present all year round [24]. To comply with the general terminology of flu surveillance, we still use the terms flu season and non-flu season, but define flu season as the mostly likely time period when flu activity peaks every year, based on literature review and official definition [19]. January to March and July to August are then defined as flu season, while the remaining periods are referred as non-flu season. The sole exception is the summer of 2007 when the flu activity in multiple data streams started peaking before July; the flu season is therefore readjusted to begin at week 25 instead of week 27 of 2007. The non-pandemic period in this model is defined as the first 120 weeks starting from the first week of 2007 until the 16^th^ week of 2009 (January 1^st^ 2007 to April 19^th^ 2009), given the fact that the U.S. Centers for Disease Control and Prevention (CDC) first announced the novel H1N1 virus alert on April 21^st^, during the 17^th^ week [25]. The pandemic period starts at week 25 and ends at week 47 of 2009 (June 15^th^ 2009 to November 22^nd^ 2009), which is when the reliable estimates of incidence rate are available.

### Statistical model

#### Bayesian hierarchical model

Bayesian hierarchical modelling framework allows for decomposing complex problems into subset of simpler problems governed by simple rules of probability [26]. Hierarchical modeling has many advantages such as allowing for multiple sources of data and accounting for parameter uncertainty; in particular, the Bayesian framework allows for the ability to consider scientifically meaningful structures and *a priori* knowledge regarding the model parameters.

Given the availability of reliable estimates of incidence rate of influenza during the pandemic period, we applied two models in this study.

#### Pandemic Model (P Model)

For pandemic period, a model is constructed based on the hypothesis that, the values reported by each surveillance system reflect both actual disease status and public awareness. The information environment input can influence the level of actual disease trends being captured through a multiplier *θ*_*j*,*t*_, and the level of public awareness imbedded in the surveillance systems *φ*_*j*,*t*_ for each surveillance system *j* at time *t*.

For data as proportions in the pandemic model (P model) model we have…

Data model:
1

Process model:
234

Parameter model:
567

For data as counts in P model we have…

Data model:
8

Process model:
91011

In P model, *X*_*t*_ denotes as the estimated influenza incidence rate of the whole population; *Y*_*j*,*t*_ refers to the data from surveillance system *j* at time *t*. The log of *Y*_*j*,*t*_ follows a normal distribution with mean of *μ*_*j*,*t*_ and variance of . *μ*_*j*,*t*_ has two components: *θ*_*j*,*t*_ ― denoted as “completeness”, describes the component of actual disease trends that surveillance system *j* captures at time *t* which is further regressed on predictive variables of the information environment proxy data (); *φ*_*j*,*t*_ ― denoted as “excess”, estimates the component in the surveillance data that cannot be fully explained by the actual disease trends, and is regressed on another set of information environment predictors. *β*_*j*,*t*,*m*_ and *α*_*j*,*t*,*n*_ are the coefficients for the information environment proxy data (*k*^*p*^) during the pandemic period. The parameter model, as stated in equation (5) and (6), apply to both models for data as counts and proportions.

#### Non-pandemic (NP) model

Due to the lack of estimated incidence rate during the non-pandemic period, a different model is constructed to assess the statistical relationship between surveillance data and the information environment. A linear regression model is fitted as follows:

Data model:
12

Process model:
13

Parameter model
1415

where *Y*_*j*,*t*_ refers to the data from surveillance system *j* at time *t*; *s* refers to either flu season or non-flu season; and *np* denotes non-pandemic period. *Log*(*Y*_*j*,*t*_) follows a normal distribution with mean *μ*_*j*,*t*_ and constant variant .  (*i* = 1,2,3) are the three information environment indices during non-pandemic period as described in Table 2. *ρ*_*s*,*j*,*t*,*i*_ is the indicator of the correspondence between the expected values of the log of the surveillance data *μ*_*j*,*t*_ and each of the *k* terms.

**Table 2:** **List of information environment indices used in pandemic and non-pandemic model**

Parameter	Coefficient	Acronym	
	*β*_*j*,*t*,2_	Sea.flu	Google search index for seasonal flu terms
	*β*_*j*,*t*,3_	Symp	Google search index for symptoms
	*β*_*j*,*t*,4_	Med	Google search index for medications
	*β*_*j*,*t*,5_	Non-flu	Google search index for non-flu terms
	*α*_*j*,*t*,2_	Total	HealthMap total number of alerts
	*α*_*j*,*t*,3_	Unique	HealthMap number of unique alerts
	*α*_*j*,*t*,4_	HCF	HealthMap number of healthcare facilities related alerts
	*α*_*j*,*t*,5_	%RSV	Lab surveillance% RSV tested positive
	*α*_*j*,*t*,6_	Authority	Google search index for authority
	*α*_*j*,*t*,7_	Pan.flu	Google search index for pandemic flu terms
	*ρ*_*s*,*j*,*t*,2_	Non-flu Index	Avian influenza, bird flu, common cold, severe acute respiratory syndrome (SARS), pneumococcus,% RSV
	*ρ*_*s*,*j*,*t*,3_	Illness Index	body temp,% RSV, cough, fever, nasal congestion, cough relief remedies, headache, common cold, pediatric, paediatric
	*ρ*_*s*,*j*,*t*,4_	Public-awareness Index	CHP, Hospital Authority, Ministry of Health, disinfect, flu, influenza, pandemic, epidemic, school closures, total number of HealthMap alerts, number of healthcare facilities related alerts, number of school related alerts, number of “breaking” alerts

Non-informative priors were used for both pandemic and non-pandemic models. A normal distribution with mean of 0, variance of 100 (or precision of .01) was used as prior distribution for *α*_*j*,*t*,*n*_, *β*_*j*,*t*,*m*_ and *ρ*_*s*,*j*,*t*,*i*_. A Gamma distribution with mean 1 and variance of 10 or Gamma(.01, .01) was used as prior for .

We conducted variable selection using a commonly used measure called the Deviance Information Criterion (DIC) as our primary method [27]. To confirm the DIC results, we also looked at other methods, such as singular value decomposition (SVD) and root-mean-square error (RMSE), which both agree with the DIC results. The predictor variables selected for the final model are listed in Table 2. Further information on model selection is available in the Additional file 1: Table S4.

Exploratory data analysis was conducted using Stata [28] and R (the Comprehensive R Archive Network) [29]. Both NP and P model are implemented in OpenBUGS [30], an open-source software package for performing Bayesian inference using Gibbs sampling. In OpenBUGS, precision *τ* (1/variance) is used to define the distributions; the posterior results are also the estimation for the precision.

Model implementation includes the choice of priors, initial values, sampling procedures, and so forth. Non-informative priors were used for both pandemic and non-pandemic model. A normal distribution with average of 0, variance of 100 was used as prior for *α*_*j*,*t*,*n*_, *β*_*j*,*t*,*m*_ and *ρ*_*s*,*j*,*t*,*i*_. A Gamma distribution (.01, .01) which has mean 1 and variance 100 was used as prior for . Using Markov Chain Monte Carlo (MCMC), we estimated the posterior distributions and reported means and standard deviations as well as 95% credible intervals (CIs) for each of the posterior distributions. In order to make sure the algorithm is robust to the choice of initial values and convergence is achieved, we used three sets of initial values. It should be noted that OpenBUGS has a feature to generate random initial values — it is advised that the user chooses the initial values carefully, as randomly generated initial values may result in epidemiologically unreasonable prior densities or unreasonable collection of values for the posterior or the likelihood function, or very slow convergence for the algorithm. The initial values used in the model, along with the OpenBUGS code, are included in Additional file 2. For each parameter, 500,000 iterations are conducted; the posteriors are calculated after the first 5,000 iterations are discarded. Three chains converge quickly for both models ― usually stabilizing after 500 iterations. 500,000 iterations with three initial chains take about 3,000 seconds for the NP model and 15,000 for the P model. No thinning is applied in either model because autocorrelation is negligible.

**Figure 2:**
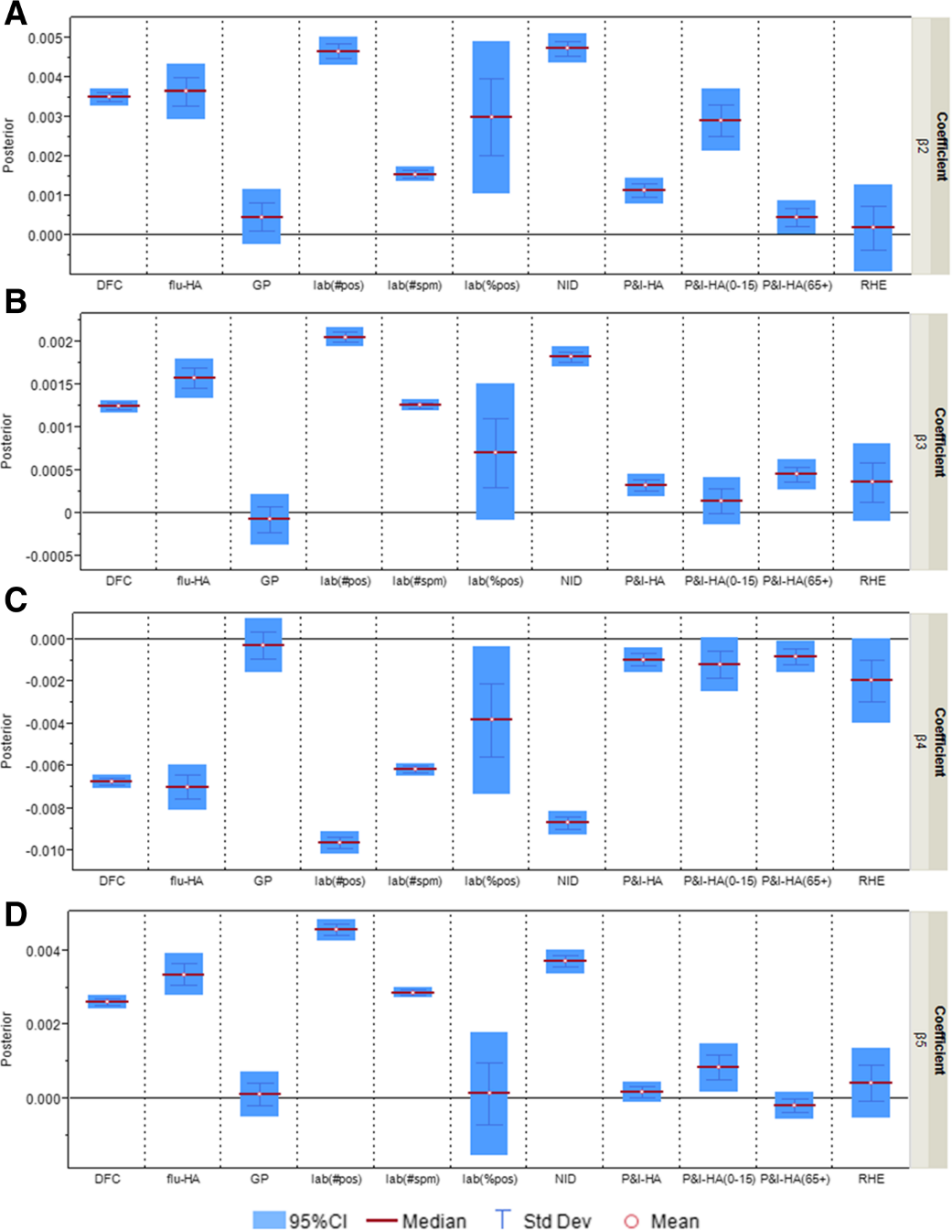
**Posterior distributions of coefficient** ***β***_***j***,***t***,***m***_ **(** ***m*** **= 2,..,5;** ***j*** **= 1,…,11) in “completeness” parameter** ***θ***_***j***,***t***_ **(multiplier for the estimated incidence rate) as measure of correspondence between surveillance data and the information environment proxy data in the pandemic model. A)** Coefficient for Google search index for seasonal flu terms; **B)** Coefficient for Google search index for symptoms; **C)** Coefficient for Google search index for flu medications; **D)** Coefficient for Google search index for non-flu terms.

To present these results graphically with 95% credible intervals (CIs), we estimate the posterior distribution of each coefficient for the correspondence between individual surveillance system and the information environment proxy data. Following standard practice in the Bayesian literature, we use the term “significant” when the 95% CI does not include zero, indicating evidence of a statistical correlation between the surveillance system and the information environment proxy data. For instance, in Figure 2A, flu-HA has a CI that is entirely above zero, which suggests a positive correspondence with one of the information environment proxy data streams — Google search for seasonal flu term. On the other hand the data for %ILI visits at general practitioners shows a lack of statistical correspondence with seasonal flu term searches because the CI includes zero.

To examine the sensitivity of the model to the choice of prior, models are run with three sets of hyper-parameters, as listed in Additional file 1: Table S7. No evidence showing sensitivity issues about the choice of hyper-parameters was observed.

## Results

### Pandemic model (P Model)

**Figure 3:**
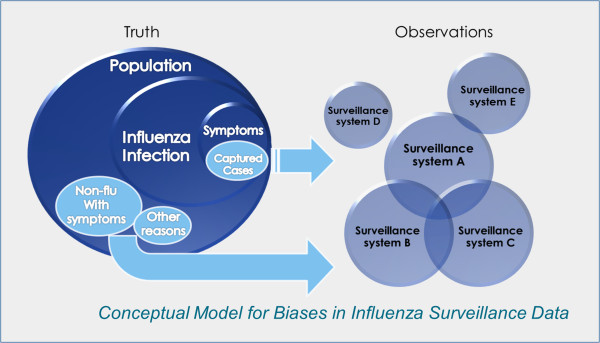
**Conceptual model for biases in influenza surveillance data.**

In the pandemic model we characterized the relationship between surveillance data and the information environment proxy data by estimating the “completeness” and “excess” in each surveillance data stream (Table 3). Figure 3 illustrates how biases can be introduced into the surveillance systems by drawing in not only non-flu patients but also increased numbers of flu patients, who otherwise might not present themselves to any of the surveillance systems. The proportion of the infected population captured by the surveillance systems — defined as “completeness” — is usually not constant but fluctuates. A surveillance system might only capture a small proportion of the actual infected population. However, as long as the ratio stays constant and independent from the information environment, it can be a useful for tracking disease in the population.

Out of eleven surveillance data streams, five — flu-HA, percentage of ILI visits at designated flu clinics, notifiable infectious disease reporting, number of specimen tested positive, number of specimen received — have consistently significant coefficients for all four k’s (Figure 2). In other words, the proportion of actual infected cases being captured by these surveillance systems is strongly affected by the information environment. If the coefficient has a positive CI such as Google search term for seasonal flu term (Figure 2A), it suggests more infected patient would be captured by the surveillance systems when web searching for “flu” and “influenza” increases. When the coefficient has a negative CI, such as Google search terms for flu medications (Figure 2C), it suggests that web searching related to flu medication is inversely correlated with the portion of flu infected population being captured. The category “medications” includes over-the-counter medications for ILI, which can be an indicator of self-diagnosis and self-treatment. Self-treatment behavior might be related to the decision of not seeking medical attention from healthcare practitioners, and therefore inversely correlated to the flu hospitalization, general practitioner patient visits, and so forth.

**Figure 4:**
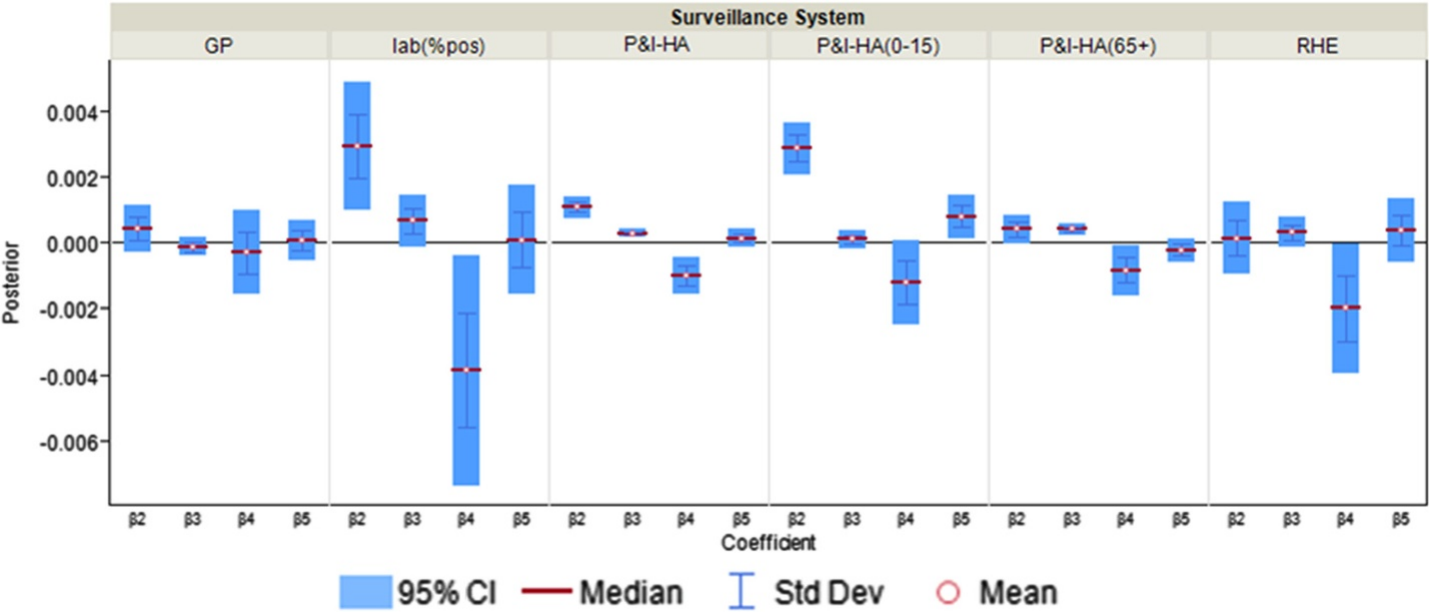
**Posterior distributions of “Completeness” coefficient** ***β***_***j***,***t***,***m***_ **(** ***m*** **= 2,..,5;** ***j*** **= 4,…,9) in “completeness” parameter** ***θ***_***j***,***t***_ **(multiplier for the estimated incidence rate) as measure of correspondence between surveillance and the information environment proxy data for surveillance systems that are less correlated with the information environment during the pandemic period.** β2 is the coefficient for Google search index of seasonal flu terms; β3 is the coefficient for Google search index for symptoms; β4 is the coefficient for Google search index of medications; β5 is the coefficient for Google search index of non-flu terms.

The remaining six surveillance data series — percentage of ILI visits at general practitioners, fever surveillance at residential homes for the elderly, P&I-HA, P&I-HA(0-15 yr), P&I-HA(65+ yr), and the percentage of specimen tested positive — have more than one insignificant coefficient for the correspondence with the information environment proxy data (Figure 4). When the coefficient is insignificant, such as Google search term for seasonal flu (Figure 2A), only general practitioners, residential homes for the elderly and P&I-HA(65+ yr) data series have coefficients that are insignificant, that is, are tracking the actual disease trends at a constant rate unrelated to the information environment.

**Table 3:** **Summary of Pandemic and Non-pandemic Model**

Data Model	
❖ Data as counts	*Y*_*j*,*t*_∼*Pois*(*λ*_*j*,*t*_)
❖ Data as a proportion	
Process Model	
❖ Pandemic Model	
▪ Data as counts	*μ*_*j*,*t*_ = *θ*_*j*,*t*_ ⋅ *X*_*t*_ + *φ*_*j*,*t*_
▪ Data as a proportion	*Log*(*λ*_*j*,*t*_) = *θ*_*j*,*t*_ ⋅ *X*_*t*_ + *φ*_*j*,*t*_
▪ “Completeness”	
▪ “Excess”	
❖ Non-pandemic Model	
▪ Data as counts and as a proportion	
	
Parameter Model: non-informative priors	
❖ Pandemic Model	
	

❖ Non-pandemic Model	
	*τ*∼*Gamma*(*a*, *b*)

**Figure 5:**
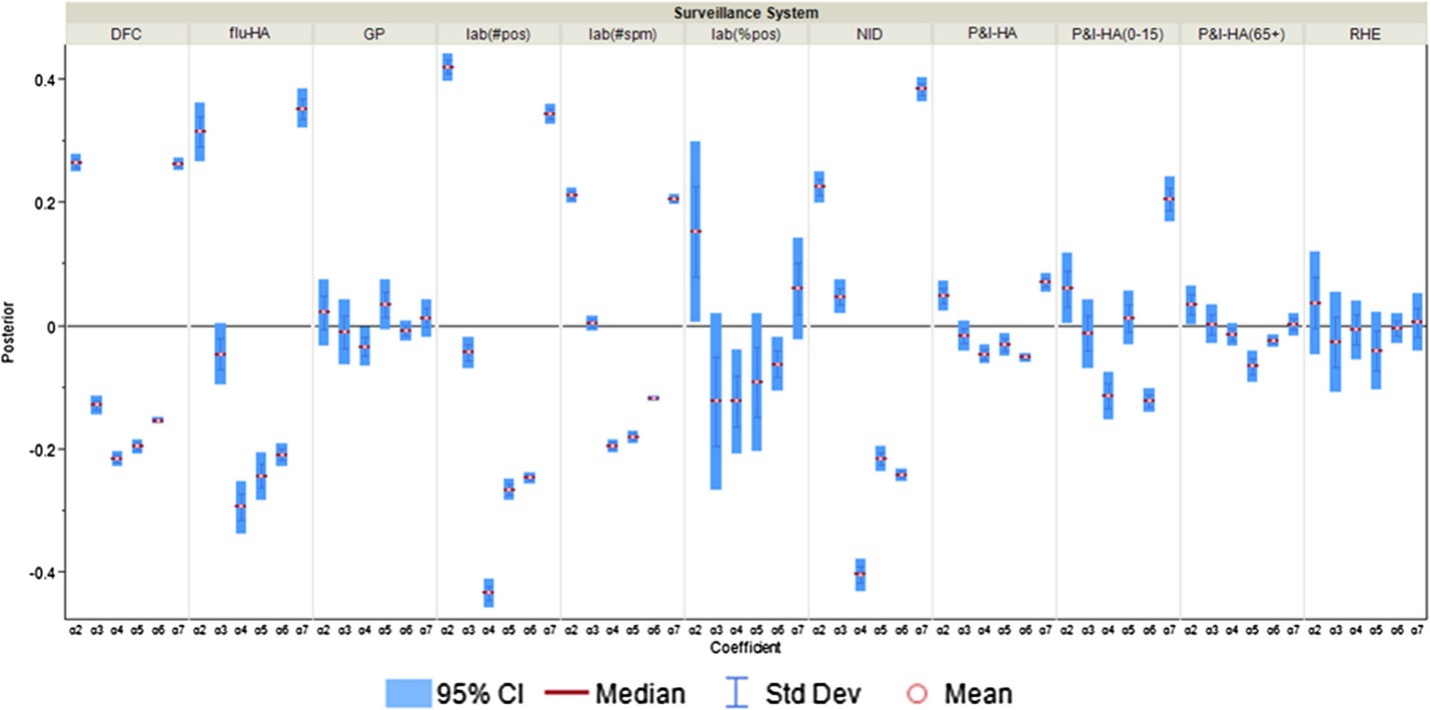
**Posterior distributions of** ***α***_***j***,***t***,***m***_ **(m = 2,..,7; j = 1,…,11) in “excess” parameter** ***φ***_***j***,***t***_ **as measure of correspondence between surveillance and the information environment proxy data during the pandemic period.** α2 is the coefficient for total number of alerts at HealthMap; α3 is the coefficient for the total number of unique alerts at HealthMap; α4 is the coefficient for number of healthcare facilities related alerts at HealthMap; α5 is the coefficient for %RSV from virological surveillance; α6 is the coefficient for Google search index of authority; α7 is the coefficient for Google search index of pandemic influenza terms.

**Figure 6:**
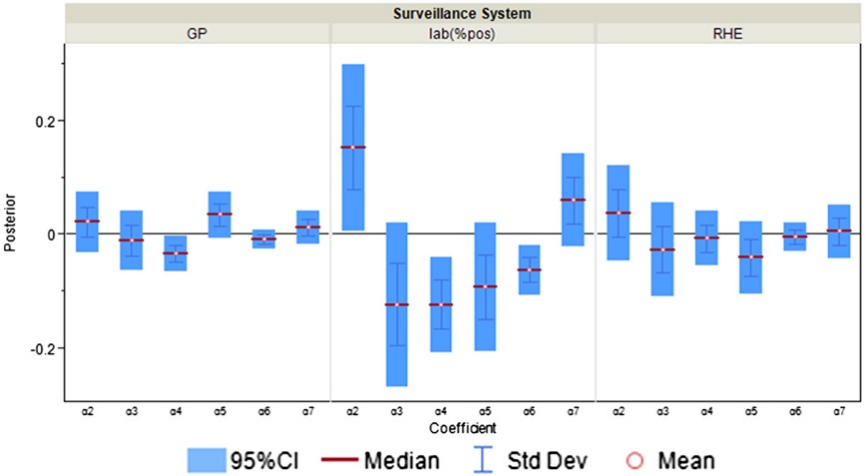
**Posterior distributions of** ***α***_***j***,***t***,***m***_ **(** ***m*** **= 2,..,7;** ***j*** **= 4,5,6) in “excess” parameter** ***φ***_***j***,***t***_ **as measure of correspondence between surveillance and the information environment proxy data for surveillance systems that are less correlated with the information environment during the pandemic period.** α2 is the coefficient for total number of alerts at HealthMap; α3 is the coefficient for the total number of unique alerts at HealthMap; α4 is the coefficient for number of healthcare facilities related alerts at HealthMap; α5 is the coefficient for %RSV from virological surveillance; α6 is the coefficient for Google search index of authority; α7 is the coefficient for Google search index of pandemic influenza terms.

“Excess,” parameterized as *φ*_*j*,*t*_, is meant to account for the variability in the surveillance data that cannot be explained by incidence rate only. In the original conceptual model, *φ*_*j*,*t*_ describes the non-flu cases captured in the surveillance systems. However, since the model itself does not have any mechanism to distinguish flu cases from non-flu cases, *φ*_*j*,*t*_ is then interpreted as the overall biases in the surveillance systems due to public awareness of the disease. The parameters *α*_*j*,*t*,2_ to *α*_*j*,*t*,7_, which can be interpreted as reflecting excess reported cases, are mostly significant for %ILI visits at designated flu clinics, notifiable infectious diseases reporting, the number of specimens received, and the number of specimens tested positive. In other words, these surveillance data streams are more likely to show an increase parallel to the information environment. On the other hand, general practitioners and residential homes for the elderly data series (Figures 5 and 6) are less likely to reflect excess cases. Among the hospitalization data series, P&I-HA(65+ yr) has insignificant coefficients for total number of alerts, number of unique alerts, and number of healthcare facilities alerts on HealthMap as well as Google search terms for pandemic influenza, suggesting that they are unrelated to the information environment. On the other hand, flu-HA has significant coefficients for almost all k’s (Figure 6). Posterior distributions for all coefficients can be found in Additional file 1: Table S8.

### Non-pandemic model (NP Model)

**Figure 7:**
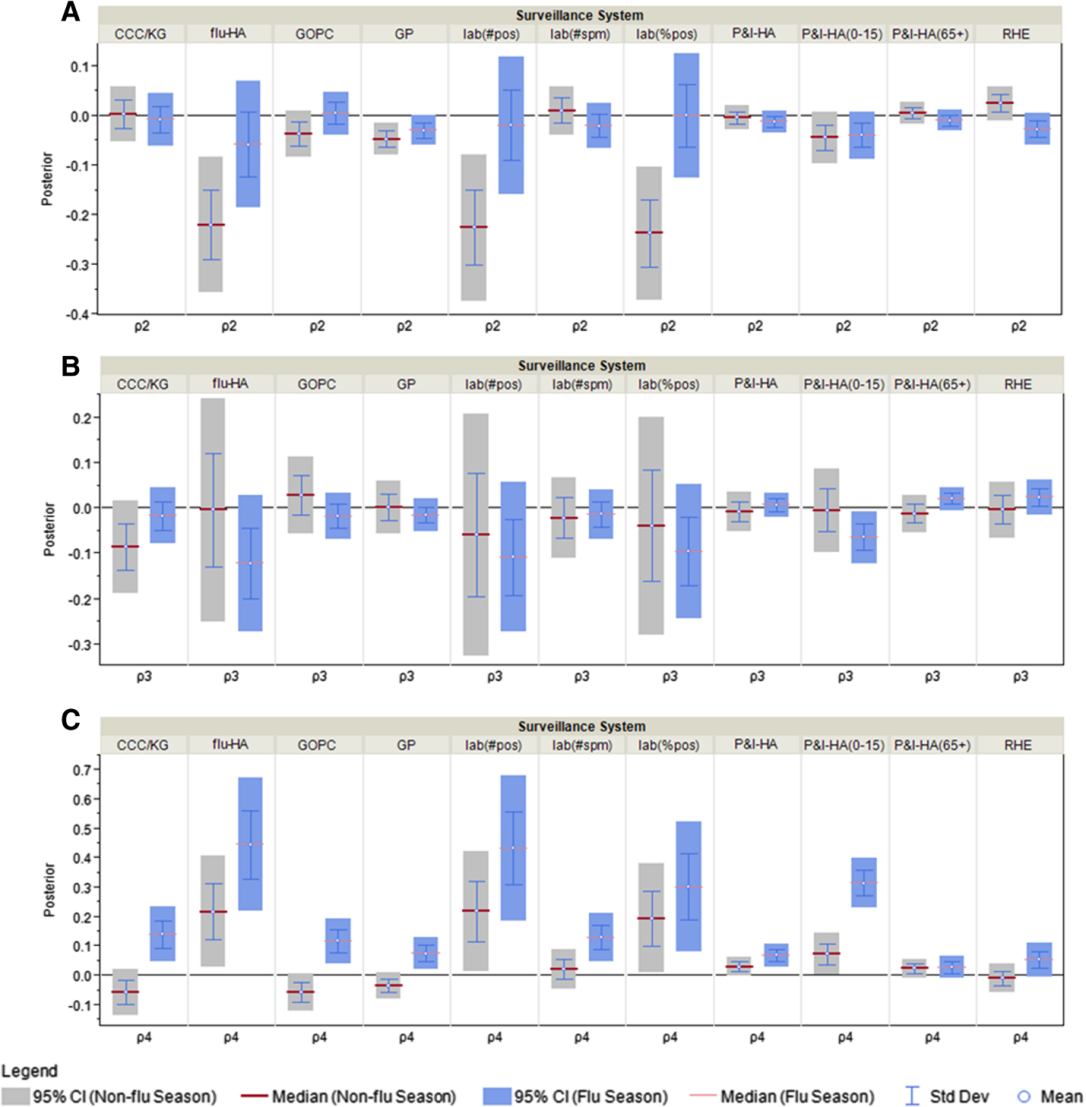
**Posterior distributions for the public awareness index and non-flu index coefficient as measure of correspondence between surveillance and the information environment proxy data during the non-pandemic model. A)** ρ2: coeffcieint for non-flu index in the NP model; **B)** ρ3: coefficient for illness index in NP model; **C)** ρ4: coeffcieint for public awareness index in the NP model.

During the flu season all surveillance data streams except the P&I HA (65+ yr) and residential homes for the elderly have significant coefficients for the public awareness index (Figure 7C), or in other words, are highly correlated with the public awareness of respiratory diseases. For sentinel surveillance systems at general out-patient clinics and general practitioner and children-specific surveillance systems at kindergarten/daycare centres and hospitals (P&I-HA (0-15 yr)), the differences between flu season and non-flu season are significant, which suggests these surveillance systems behave differently with respect to public awareness in the flu and non-flu seasons. The non-flu index coefficient is significant for flu hospitalization, number of lab tested positive specimens, percentage of specimens tested positive and percentage of ILI-visits at general practitioners during the non-flu season, which suggests these surveillance data streams are influenced by the information environment related to other diseases such as common cold when flu activity is low. During the flu season, however, the coefficient for non-flu index is insignificant for all surveillance systems (Figure 7A), which suggests none of the surveillance data streams is influenced by non-flu related information environment.

**Figure 8:**
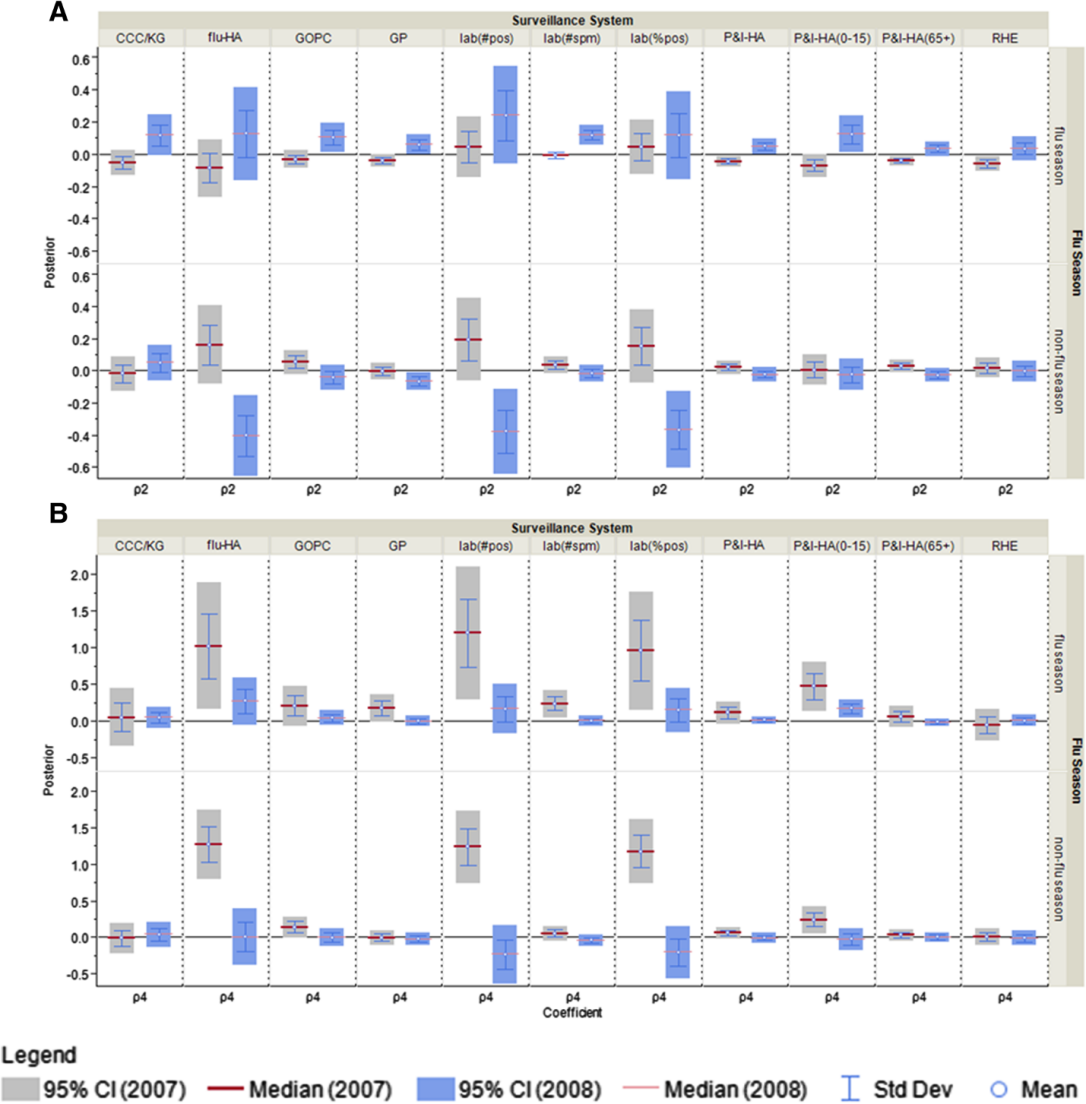
**Comparison of posterior distributions for the public awareness index and non-flu index coefficient as measure of correspondence between surveillance and the information environment proxy data in 2007 and 2008. A)** ρ2: coeffcieint for non-flu index in the NP model; **B)** ρ3: coeffcieint for public awareness index in the NP model.

**Figure 9:**
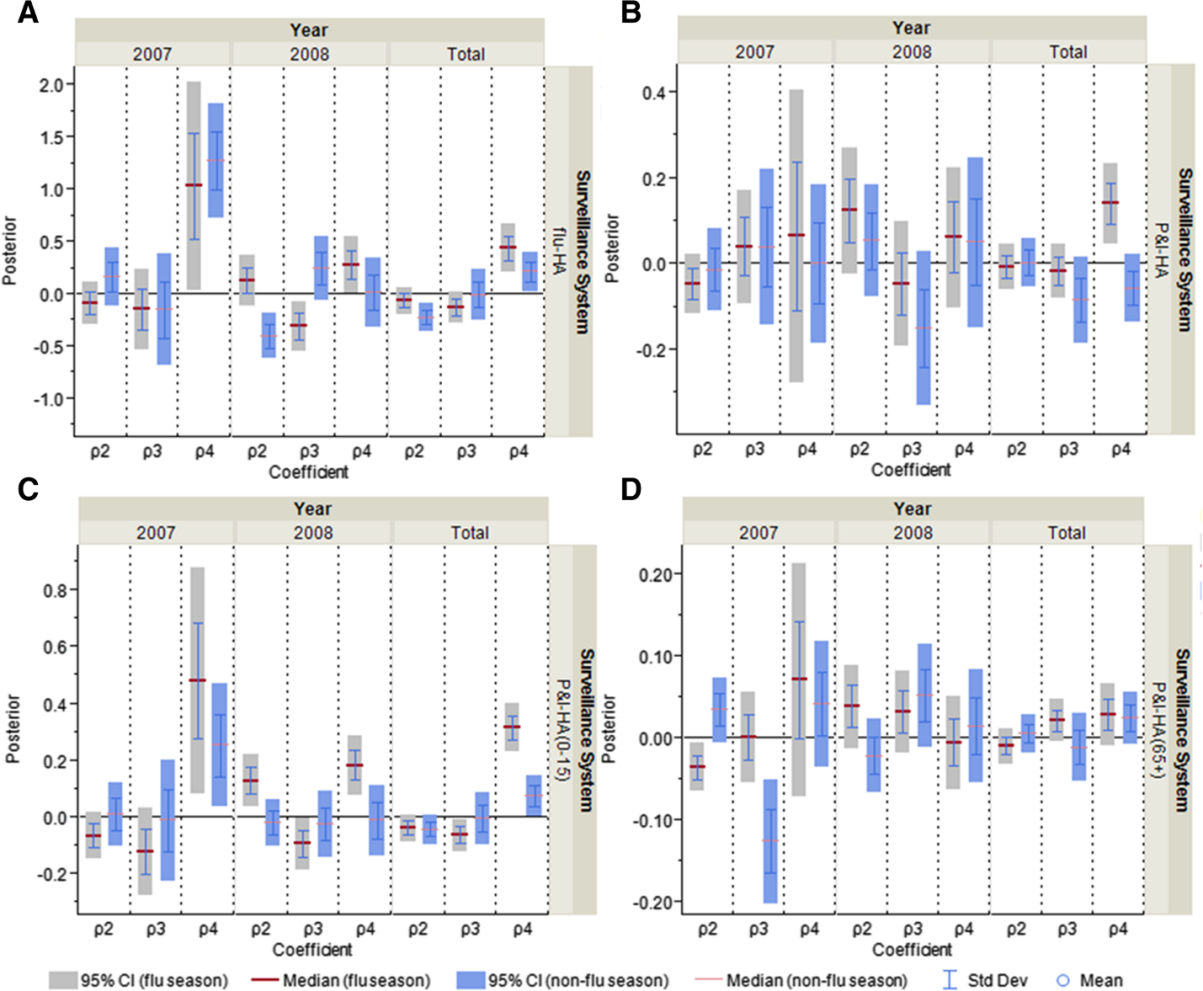
**Comparison of posterior distributions for non-flu index, illness index and public awareness index as measure of correspondence between surveillance and the information environment proxy data for hospitalization data series during the non-pandemic period. A)** flu-HA; **B)** P&I-HA; **C)** P&I-HA(0-15 yr); **D)** P&I-HA(65+ yr).ρ2 is the coefficient for non-flu index, ρ3 is the coefficient for illness index, and ρ4 is the coefficient for public awareness index.

When the non-pandemic period data is divided into 2007 and 2008, and the model fit separately for each period, two sets of posteriors ― 2007 (week 1-52) and 2008 (week 53-104) are compared. For public awareness index coefficient, the heterogeneity between 2007 and 2008 is less obvious during the flu season as compared to non-flu season (Figure 8B). In other words, the correspondence between the surveillance data streams and public awareness is relatively stable from year 2007 to 2008 when flu transmission is active. For the non-flu index (Figure 8A), during the flu season, P&I-HA, P&I-HA(0-15 yr) and lab(#spm) have significantly different coefficients, which suggests when the flu activity is high, the increased reports of P&I-HA and pediatric P&I-HA, plus the number of specimens sent to the lab is correlated with the information environment related to non-flu respiratory diseases. As shown in Figure 9, when comparing the four hospitalization data streams side by side, flu-HA looks more similar to P&I-HA(0-15 yr), and P&I-HA is more similar to P&I-HA(65+ yr).

Posterior distributions for all coefficients in NP model can be found in Additional file 1: Table S10.

## Discussion

In their efforts to develop new methods for influenza surveillance, researchers have considered many different data sources, most of which already exist in electronic form. Some derive from traditional surveillance approaches while using influenza-like-illness (ILI) and other data that do not require laboratory diagnosis. Others, such as the Global Public Health Intelligence Network (GPHIN) and HealthMap, search results from the Internet and other media sources via automated algorithms to identify disease outbreaks that might not have been recognized by the authorities [3, 4]. Google Flu Trends uses influenza-related search queries to model flu activity [31], while some other studies try to capture ILI through micro-blogging platforms such as Twitter [32]. New terms such as “Internet-based surveillance”, “digital disease detection”, and “inforveillance” have been introduced to describe such public health surveillance practices [20, 10].

Recent studies have found a high correlation between syndromic surveillance and traditional influenza surveillance data [33–38]. Internet-based surveillance such as Healthmap and Google Flu Trends have claimed success in capturing pandemic flu outbreak [3, 4] and tracking flu activity [11] days to weeks ahead of standard Centers for Disease Control and Preventions (CDC) systems. With its advantages of timeliness and low cost, Internet-based surveillance systems have been widely recognized as important supplementary data sources and widely used as the baseline standard for evaluating new influenza surveillance systems [5, 39–41].

Internet-based surveillance data, however, reflects both a “signal” reflecting actual disease trends and “noise” caused by changes in public awareness. How to accurately and effectively separate the “signal” from the “noise” becomes one of the biggest challenges in analysing internet-based surveillance data. Some researchers have developed natural language processing algorithms to classify this information automatically [34, 42], some use crowd-sourcing platforms to engage Internet users in tagging data manually [43], and some use both [44]. The curated data, which is thought of as reflecting actual disease status, is then compared to the traditional surveillance data and tested for correlations [31, 32, 34, 35]. This approach, however, does not prove the validity of the new surveillance method since both data streams may reflect the same information environment, therefore be biased in the same way. For instance, Google Flu Trends, which had been performing well in tracking CDC surveillance data, dramatically over-estimated the flu activity in the United States in 2012-13 flu season [37]. The over-estimation might be due to the extensive media coverage of flu during the winter holiday season [45], but raises the question of how well Internet-based surveillance systems reflect flu activity per se, rather than other factors such as public awareness.

Our analysis uses a Bayesian hierarchical statistical model to estimate the correspondence between individual surveillance data and the information environment proxy data. The model structure is developed based on an understanding of disease surveillance being a process rather than a direct reflection of disease status per se. The statistical model does not directly describe disease transmission dynamics, and the goal is not to estimate parameters or flu activity level. Rather, as a characterization tool, this analysis reveals how surveillance systems “behave” differently under changing information environments. Similarly, the purpose of model fitting is not to identify the perfect model with the best fit for the data. Rather, the goal is to find a model that captures the relationship between the surveillance systems and the information environment that is consistent with epidemiological expertise and practitioners’ understanding of the actual disease process, and thus one that is likely to be applicable in the future.

Among all the influenza surveillance data that we studied, we found some surveillance systems that more consistently corresponded to the information environment proxy data than others. The level of correspondence with the information environment is associated with certain characteristics of the surveillance data. General practitioner (%ILI-visit) and laboratory (%positive) seem to proportionally reflect the actual disease trends and are less representative of the information environment. Surveillance systems using influenza-specific code for reporting tend to reflect biases of both healthcare seekers and providers. This pattern is what we would expect to see if the information environment were influencing the observable data.

### Characterization of surveillance systems using the pandemic model

When looking at “completeness” only, three types of surveillance systems show a certain level of stability in the changing information environment. Surveillance data in percentages such as percentage of specimen tested positive, percentage of ILI visits at general practitioners and percentage of fever at residential homes for the elderly tend to have insignificant CIs for “completeness.” Surveillance systems that use less specific diagnostic and reporting codes such as “pneumonia and influenza hospitalization” and “fever at residential homes for the elderly” are also less likely to be influenced by the search index and the media coverage. Surveillance systems monitoring the elderly tend to be less susceptible to the information environment as compared to those monitoring children, which can be observed by the comparison between P&I-HA(0-15 yr) and P&I-HA(65+ yr) (Figures 4 and 5). For surveillance systems that meet more than one criterion, the correlation with the information environment is weaker than those only meet one.

Surveillance data represented as percentages seem to be less correlated with the information environment, perhaps because the nominator and denominator change in the same direction in response to the information environment. Reflecting general practitioners’ role as the gatekeeper of the healthcare system, general practitioner visits are predominantly influenced by only one layer of decision-making ― patients seeking medical attention. Since patients usually do not have the ability to distinguish influenza from other viral respiratory infections themselves, flu and non-flu infections may be just as likely to be presented to general practitioners. This pattern might not hold during the early stage of a pandemic, when the spread of novel influenza virus may not keep up with the spread of awareness, possibly leading to a negative correlation between the percentage of ILI-related general practitioner visits and the information environment. However, due to insufficient data volume, the model failed to run when segmenting the pandemic period into the early (summer) and late (fall) stage.

Percentage of specimen tested positive, on the other hand, is often used as the “gold standard” for influenza surveillance. As a surveillance system with a specific case definition based on confirmed virological testing, as well as being in a percentage format, the percentage of specimens tested positive is likely to provide the most reliable estimates of flu activity. However, an individual case has to go through at least two layers of decision-making ― patient’s decision on healthcare seeking and physician’s decision on sampling and diagnosis. Thus, it is possible that when the physicians are “sensitized” by the media and official guidelines, they may actively look for cases that fit the clinical definition of influenza and sample them for laboratory testing. This effect is more obvious in the count data for flu-HA, but may also influence the percentage of specimen tested positive as shown in Figures 2, 4 and 5.

Another pattern is the difference between influenza specific and non-specific surveillance systems. Flu hospitalization data seem be more correlated with the information environment as compared to pneumonia and influenza together. As discussed above, once sensitized, physicians are more likely to take samples from patients, and to use diagnostic codes that are specific for influenza, especially in the subpopulations that are considered to be more vulnerable to the pH1N1 virus during the flu pandemic. During the early stage of the pandemic, children and young adults were considered to be more susceptible to the novel influenza virus, which might contribute to the observation in our study that the pediatric P&I-HA tends to be more correlated with the information environment compared to the elderly.

We also observed a difference in the level of correspondence to the information environment between surveillance systems that monitor elderly versus other age groups. One possible explanation is disparities in information literacy and access to computers and the Internet among different age groups. Google searches are likely to be driven by subpopulations of specific demographic characteristics and socio-economic status, such as young and middle-aged people who have easy access to digital devices as an information portal, as compared to the elderly who live in residential homes. Although children may have limited information literacy and access, their parents are likely to take immediate action in response to the information related to children’s health.

As for the “excess” parameter, *φ*_*j*,*t*_, ILI visits at general practitioners, percentage of specimen tested positive and percentage of fever at the residential homes for the elderly show the least significant correlation with the information environment proxy data, including the number of total HealthMap alerts, unique alerts, healthcare facilities related alerts, lab(%RSV), search index for authorities and pandemic flu terms (Figure 6). The lack of significant correlation might be due to the percentage format of these data streams, since data in counts usually have significant CIs. The coefficients for the search terms for pandemic influenza are all positive for the surveillance systems represented as counts, which suggests a positive correlation between the biases in those surveillance systems and public awareness of pH1N1.

In general, the fewer layers of decision-making, the less correlated the surveillance system is with the information environment. The traditional “gold standard” surveillance systems, such as hospitalization and virologic surveillance, are subject to the biases introduced by healthcare professionals. The more specific and *ad hoc* the diagnostic and reporting codes are, the more likely it is influenced by the information environment. Surveillance data in percentage format tends to capture actual disease trends in constant ratio, less influenced by the information environment than data in counts.

### Characterization of surveillance systems using the non-pandemic model

For the non-pandemic period we developed three indices to describe the relations between surveillance data and information environment proxy data — an actual disease status indicator (illness index) plus public awareness of both influenza and other viral respiratory diseases (public awareness index and non-flu index). Since we are most interested in the correspondence between the surveillance data and the public awareness index, the posterior distribution of the public awareness index coefficient is compared among different surveillance data streams, segmented by flu/non-flu season and year.

The public awareness index used in the NP model is a collection of search keywords and categories of HealthMap alerts that are most likely to be associated with public awareness of influenza outbreaks, such as search volume for influenza outbreaks and the number of alerts of school-based outbreaks. Surveillance systems are in general more correlated with public awareness during the flu season as compared to the non-flu season. When observing increasing flu activity in the community or from news media, one may get sensitized and tend to seek medical attention when feeling sick. The exceptions are two surveillance systems that monitor predominantly the elderly ― flu surveillance at residential homes for the elderly and P&I-HA (65+ yr) (Figure 7B). These two surveillance systems are relatively less correlated with the public awareness in most cases, and show more stability from year to year (Figure 8B). During the non-flu season, the majority of surveillance systems seem not to be influenced by public awareness except for the flu associated hospitalization, number of specimens tested positive, percentage of specimens tested positive, and P&I-HA(0-15 yr) (Figure 7B), which can also be observed in the year to year comparison graph (Figure 8B).

Beyond comparing the correlation between surveillance and information environment proxy data individually, we also made an exploratory effort to assess the similarity among different surveillance systems by using the characterization tool and the identified evidence of potential biases in clinical practice. When comparing the four hospitalization data streams side by side, we observed that flu-HA looks more similar to P&I-HA(0-15 yr) (Figures 9A and C), while P&I-HA is more similar to P&I-HA(65+ yr) (Figures 9B and D). The patterns are consistent in flu and non-flu season and in different years (Figures 8 and 9), and correspondent to the pandemic model.

In the pandemic model, we observed that flu-HA is more correlated with the information environment than P&I-HA, while pediatric P&I-HA also seems to be more correlated with the information environment than the P&I-HA for the elderly. Also, when replaced the incidence rate of all-age with 5-14 yr, data fits better for flu-HA, general practitioner ILI-visits, notifiable infectious disease reporting and P&I-HA(0-15 yr) (Additional file 1: Table S9). During the pandemic flu outbreak, since the children and young adults were considered to be at higher risk than the elderly, physicians may tend to order more laboratory testing for pediatric patients [7]. During the non-pandemic period, the same clinical practice might still prevail. Since pediatric mortality is a reportable condition, clinicians are more likely to order laboratory tests and use a specific diagnostic code if the test results are positive. The elderly patients, however, who usually have non-specific clinical manifestation for respiratory diseases and lower viral loads [46], are less likely to be sampled, less likely to have a positive result if tested, and usually given a less specific diagnostic code such as “pneumonia and influenza”.

It is worth noting that the data volume for flu-HA and pediatric P&I-HA are both relatively low during the non-pandemic period, which might also contribute to the similarity between the two data streams. Also, there is a much lower Google search volume and fewer HealthMap alerts in 2007 compared to 2008, possibly due to the introduction of smartphone and rapid growth of the Internet itself from 2007 and onwards [47].

Before the 2009 pandemic flu outbreak, the age-stratified flu-HA was not collected in Hong Kong, therefore it is difficult to test our hypothesis of the biases in clinical practice. The implications of this finding are (1) flu hospitalization might not be representative for all age groups, and (2) it is important to collect age-stratified flu hospitalization data, not only for monitoring the susceptibility of the subpopulation, but also for assessing potential biases in practice.

### Re-evaluating the usage of information environment data

This study also has implications for the use of information environment data for disease surveillance. Advances in information technology have made a wide range of data available to public health researchers and practitioners, offering the promise of improving current surveillance systems, generating more sensitive and timely warnings of disease outbreak or providing more accurate estimates of disease transmission. For this potential to be realized, however, the characteristics of these new data sources must be understood before they are used in sophisticated statistical models. Olson and colleagues have suggested, for instance, that Google Flu Trends’ impressive retrospective correlation with ILI surveillance data may be a product of over-fitting by “fishing” through numerous search term combinations as part of data mining. Moreover, Google Flu Trends’ tendency to miss the beginning of an outbreak and its poor accuracy at the local level also limits its application in providing early warning and situational awareness [37]. The importance of understanding the nature of the data and the environment in which the data is generated may be overshadowed by researchers’ and practitioners’ enthusiasm for data availability (i.e. “big data”) and purely statistical patterns (often ignoring confounding variables or underlying processes).

### Limitations

The approach we used in this study is limited by availability of data, which influenced how we evaluate the model and interpret the results. Since the reliable incidence rate estimate is not available before the 2009 pH1N1 outbreak, we developed different model for non-pandemic period, the results of which are, to some extent, consistent with what have been found in the pandemic model. Google search volume for some keywords in Hong Kong is not large enough to generate a search index; or sometimes is not of the same time resolution as the weekly surveillance data. More than half of the search keywords, for instance, are only available on monthly basis. Also, we have not exhausted all the possible combinations for keywords, HealthMap alerts count, and different time lag.

Given the noisy data and the lack of disease transmission mechanism, our search for the best fitting model might have led to over-fitting. The selection and aggregation of predictors, therefore, is guided by both practical knowledge and model performance comparison, in order to achieve a balanced model version that is of relatively good fit and meaningful for practitioners to interpret. For instance, the predictors are grouped in a relatively arbitrary manner, but the selection process for the pandemic model was blinded from the results of posteriors for each parameter before the final model version was selected.

## Conclusions

In this study, we estimated the correspondence of multiple influenza surveillance data streams with indicators of the information environment, and the results suggest that most influenza surveillance data, to some degree, reflect public awareness as well as actual disease status. For instance, individuals who are aware of the on-going transmission of influenza are likely to search for information for prevention and self-diagnosis purposes, and may tend to seek medical attention once feeling sick. Thus, although it has not been recognized and studied systemically, many influenza surveillance systems may reflect changes in the information environment as well as actual disease trends. And although the data we analysed are all from Hong Kong, the underlying mechanisms are not specific to that region, so the problem may be widespread. Indeed, Zhang and colleagues and Stoto found similar patterns using less formal methods in the United States [6, 7].

Some surveillance systems seem to represent public awareness more than actual disease status. In particular, *ad hoc* surveillance systems set up during the early ascertainment of pH1N1 outbreak — such as the walk-in clinics for ILI, making pH1N1 as a new condition for notifiable infectious disease — are more correlated with the information environment than other surveillance systems that we identified in Hong Kong. Such results help us better understand and characterize influenza surveillance systems, which can be used in data interpretation, resources allocation, new surveillance systems design and implementation in order to capture a more accurate picture of disease transmission.

The study findings discussed above are consistent with our practical knowledge that traditional and syndromic surveillance systems can be influenced by the public awareness of the disease. Other than Google searches, correlation between social media (e.g. Twitter) and similar data with traditional flu surveillance data may only indicate that both are reflecting the same information environment, rather the social media data reflecting actual disease status. Often times they are not clearly distinguished; and, people readily jump to the conclusion that the information environment data can be used as a proxy for disease status. As shown in our study, such an approach has its limitations. Information environment data such as web queries and tweets in fact have dual usage, (1) as proxy for direct estimate for disease, and (2) as covariates to control the model for public awareness biases. When researchers promote the idea of using the Internet data for disease surveillance among practitioners, the differentiation was not made clearly, and sometimes is lost when being communicated to the general public. Our study provides a framework to understand how the information environment data is related to the traditional surveillance data, which will help to fine tune the usage of information environment data.

## Electronic supplementary material

Additional file 1: **Supplementary materials including data sources, data preparation, model selection, sensitivity analysis and results of posterior distribution in table format.** (DOCX 169 KB)

Additional file 2: **OpenBUGS codes for the final pandemic and non-pandemic model.** (DOCX 23 KB)
