# Supplementary material for: Characterizing Influenza surveillance systems performance: application of a Bayesian hierarchical statistical model to Hong Kong surveillance data
Source: BMC Public Health. 2014 Aug 15;14:850. doi: 10.1186/1471-2458-14-850 (PMC4246552; doi:10.1186/1471-2458-14-850)
Supplement: Supplementary file 1 — Additional file 1: Supplementary materials including data sources, data preparation, model selection, sensitivity analysis and results of posterior distribution in table format. (DOCX 169 KB) [file 12889_2014_7289_MOESM1_ESM.docx]

Additional file I

# 1 Data sources and preparation

## 1.1 Information environment proxy data

HealthMap collects disease related information in multiple languages including Chinese. However, the curation of Chinese feeds is not as good as the English feeds due to the lack of language specialists. In early preparation of the study, a complete manual curation of the Chinese news feed in the HealthMap system was carried out for alerts labeled as “respiratory illness” in disease category and “Hong Kong” in geographic location. The curation includes: 1) labeling the alert with “breaking”, “warning”, “context”, “old news”, “NDR(non-disease related)”; 2) assessing if the specific alert is unique or duplicate; 3) examining the geographic location, disease category, place category and number of cases reported. After the curation, data is extracted using the following criteria.

Among the 2,166 records retrieved from HealthMap data base from the oldest available alert to November 22, 2009. Only 16 alerts are listed from 2000 to 2007. Considering the incompleteness of the HealthMap data before 2007, the first week of 2007 is then set as the starting point for the whole model.

Table S1 Inclusion and exclusion criteria for HealthMap data

| **Inclusion criteria** | |
| --- | --- |
| Under “Advanced Search” | |
| Feed | English: ProMed, WHO, OIE, FAO, EuroSurveillance, Google News, Moreover, Wildlife Diseaes Information Node |
|  | Chinese: Baidu News, SOSO Info |
| Time | January-01-2000 to September-24-2012 |
| Place | Hong Kong |
| Under “Account-> Preferences->Preferred Feeds, Diseases & Locations” | |
| Disease category | Respiratory |
| **Exclusion criteria** | |
| Tag | NDR |

All Google search index data are standardized using the study period average search volume as denominators. And for quality control purpose, the “related terms” shown on the Google Trends website for each search keyword is also examined and documented. Some key words are significantly correlated with non-diseases related terms. For example, “N95”, also called respirator, is used as a preventive measure for contagious respiratory diseases; the same word is used for a cell phone model released during the study period. On the other hand, “common cold” in Chinese is correlated with the brand name of the most popular OTC flu medication “Calitan”, therefore included in the category of flu medications as well. In Table S2, all key words are listed under seven categories.

Table S2 Categorization of the informational environment proxy data

| Google Search Index |  |
| --- | --- |
| Category | Key words |
| General term: seasonal flu | flu, influenza, flu(*liu gan*, Chinese) |
| General term: pandemic flu | h1n1, swine flu, swine flu(*zhu liu gan*, Chinese) |
| Non-flu disease term | SARS, avian influenza, pneumococcus(*fei yan qiu jun*, Chinese), bird flu, bird flu (*qing liu gan*, in Chinese), common cold(*gan mao*, Chinese), common cold (*shang feng*, Chinese) |
| Symptoms | cough, fever, headache, body temperature, cough(*ke sou*, Chinese), sore throat, fever(*fa shao*, Chinese), headache(*tou tong*, Chinese), body temperature(*ti wen*, Chinese), nasal congestion(*bi sai*, Chinese) |
| Medications | tamiflu, cough relief(*zhi ke*, Chinese), calitan(*gan mao*, Chinese) |
| Authority | Hospital Authority, Hospital Authority ( *yi guan ju*, Chinese), Ministry of Health, Ministry of Health (*wei sheng shu*, Chinese), Centre for Health Protection, Centre for Health Protection (*wei sheng fang hu zhong xin*, Chinese), pandemic, epidemic( *yi qing*, Chinese), |
| Prevention | Disinfect ( *xiao du*, Chinese), face mask( *kou zhao*, Chinese) |
| Children related | pediatric( *er ke*, Chinese), paediatric, school closure(*ting ke*, Chinese) |
| HealthMap |  |
| General | Number of total articles, number of unique articles |
| Warning level specific | Number of articles labeled as “breaking”, number of articles labeled as “warning”, number of articles labeled as “context” |
| Location specific | Number of articles with location category of nurseries, preschools, elementary schools and secondary schools, number of articles with location category of healthcare facilities |
| Case specific | Number of articles with any of the suspicious, confirmed, death case reported. |

## 1.2 Influenza surveillance data

Data as proportions, including the estimated incidence rate, are converted to per 1,000 scales. During non-pandemic period, the missing data points in CCC/KG are estimated by using the average of the week before and after, or the weighted average if there are two consecutive data points missing.

Majority of the data series have slightly different start and end dates for a week with one or two days of mismatch. The starting date of January 1^st^ 2007, used by laboratory surveillance data, is then applied to all data streams for convenience, including the informational environment proxy data.

# 2 Model selection

## 2.1 Choice of distribution

The general principle for choosing distribution is based on the nature of the data — normal distribution is considered as default model choice for percentage data, while Poisson distribution is chosen for count data. Data symmetry is also examined from the histograms. For non-pandemic period, the histogram of all data streams, especially the count data, show improvement after log transformation, which is not observed from the pandemic period except the percentage data. Therefore Log-normal distribution is chosen for non-pandemic model, while Poisson and Log-normal mixed model is chosen for pandemic model.

For count data, negative bionomial distribution is also considered. However, when fitting the response variable to negative binomial regression model, the dispersion parameter α is not significantly greater than zero ― none of the 95% CI is over 1. Lack of evidence for over-dispersion, Poisson distribution is chosen for P model.

Table S3 Choice of distribution for each surveillance data stream

| Period | Data format | Symmetry before log transformation | Symmetry afterry before log transformationt rate, erage of the week before and after the missing week, or the weighted average if there a log transformation | Choice of Distribution |
| --- | --- | --- | --- | --- |
| Non-pandemic | Count data | Low | Improved | Log-normal |
| Non-pandemic | Percentage data | Median | Improved | Log-normal |
| Pandemic | Count data | Low | Not improved | Poisson |
| Pandemic | Percentage data | Low | Improved | Log-normal |

## 2.3 Model simulation

Simulation was conducted based on the pandemic model using the United States flu surveillance and informational environment proxy data [1]. The CDC ILINet outpatient visit data is used as the simulated incidence rate $X_{t}$, Google search index for key words: “flu”, “influenza”, “swine flu” and “h1n1”, and the number of HealthMap articles in English, under the category: “Swine flu H1N1” for the United States are used as parameters in $\varphi_{j,t}$. Single data stream in normal distribution is used for the simulation. The majority of picked values for the coefficients are recovered other than two of the intercepts are slightly outside the 95% CI of the posteriors.

## 2.3 Predictors selection

We conducted variable selection using a commonly used measure called the Deviance Information Criterion (DIC). DIC was introduced by Spiegelhalter as a generalization of Akaike's information criterion (AIC) [2], is a penalized likelihood method based on the posterior distribution of the deviance statistic defined as

$$D(\theta)=-2\log\left(p\left(y|\theta\right)\right)+C$$

where$y$denotes the data,$p\left(y|\theta\right)$denotes the sampling distribution (i.e., likelihood function) of data given model parameters (θ), and *C* is a constant.

DIC is defined as

$$DIC=2\bar{D}-D(\bar{\theta}$$

where
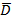
 is the posterior mean of the deviance, and
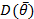
 is the deviance of the posterior mean values for the model parameters (denoted by *θ*). Based on the DIC criterion, models with relatively lower DIC values indicate a better fit to the data compared to models with higher DIC values.

For P model, three steps are taken to select the final predictive variables $k_{n,t}^{p}$ from all the data input listed in Table S2. First, $\theta_{j,t}$ is held constant, Deviance Information Criterion (DIC) and the effective number of parameters (pD) value are compared between the two models with either Google search data or HealthMap data in $\varphi_{j,t}$. And the same procedure is repeated when $\varphi_{j,t}$ is assigned a constant value. Model performance is improved when having Google search in $\theta_{j,t}$and the HealthMap data in $\varphi_{j,t}$ [3].

To further simplify the model, predictors are further assessed and selected for the final version of the model. Multi-collinearity is assessed to avoid combinations that have high collinearity among the predictors. Then starting with two predictors in both $\theta_{j,t}$ and $\varphi_{j,t}$, the combinations with the lowest DIC and pD are kept for the next round when more predictors added. Some practical knowledge is also used to inform the model selection. For instance, the search index for pandemic flu and authorities are placed in $\varphi_{j,t}$, which is likely to be associated with the public awareness of the influenza epidemic. Also, in order to estimate the influence of the co-circulating Respiratory syncytial virus (RSV), percentage of RSV tested positive from the laboratory surveillance is also added as one of the predictive variables. The DIC and *pD* values for the initial 36 models are listed in Table S4, model 36 is selected for P model.

Table S4 Model performance comparison for different set of precitors

| ID | $\boldsymbol{\theta}$ | $\boldsymbol{\varphi}$ | 1wk Lag | DIC | *pD* |
| --- | --- | --- | --- | --- | --- |
| 1 | sym,med | *total,unique,HCF*,RSV | N | 9365 | 54.42 |
| 2 | sym,med | *tag1,tag2, HCF*,RSV | N | 11410 | 53.27 |
| 3 | sym,med | *tag1,tag2,HCF*,RSV | Y | 10690 | 53.81 |
| 4 | sym,med | *total,unique,HCF*,RSV | Y | 10950 | 53.21 |
| 5 | sym,med | *total,unique,school*,RSV | Y | 13800 | 53.02 |
| 6 | sym,med | *tag1,tag2,school*,RSV | Y | 12610 | 53.56 |
| 7 | sym,med | *tag1,tag2,school*,RSV | N | 12410 | 53.69 |
| 8 | sym,med | *total,unique,school*,RSV | N | 10290 | 54.8 |
| 9 | flu terms(seasonal+pandemic), sym | *total,unique,school*,RSV | Y | 14350 | 53.52 |
| 10 | flu terms(seasonal+pandemic), sym | *total,unique,school*,RSV | N | 10980 | 54.18 |
| 11 | flu terms(seasonal+pandemic), sym | *total,unique,HCF*,RSV | Y | 10980 | 72.15 |
| 12 | flu terms(seasonal+pandemic), sym | *total,unique,HCF*,RSV | N | 9585 | 54.69 |
| 13 | flu terms(seasonal+pandemic), sym | *tag1,tag2,school,*RSV | Y | 13440 | 53.19 |
| 14 | flu terms(seasonal+pandemic), sym | *tag1,tag2,school*,RSV | N | 12960 | 53.03 |
| 15 | flu terms(seasonal+pandemic), sym | *tag1,tag2,HCF* | Y | 10390 | 72.16 |
| 16 | flu terms(seasonal+pandemic), sym | *tag1,tag2,school*,RSV | N | 11840 | 53.38 |
| 17 | nonflu,authority | *tag1,tag2,school*,RSV | N | 13190 | 52.38 |
| 18 | nonflu,authority | *tag1,tag2,HCF*,RSV | N | 12810 | 52.86 |
| 19 | nonflu,authority | *tag1,tag2,HCF*,RSV | Y | 10600 | 54.2 |
| 20 | nonflu,authority | *total,unique,school*,RSV | Y | 12840 | 51.99 |
| 21 | nonflu,authority | *total,unique,school*,RSV | N | 12430 | 53.18 |
| 22 | nonflu,authority | *tag1,tag2,school*,RSV | Y | 13400 | 52.83 |
| 23 | nonflu,authority | *total,unique,HCF*,RSV | N | 12040 | 53.03 |
| 24 | nonflu,authority | *total,unique,HCF*,RSV | Y | 10630 | 52.65 |
| 25 | flu terms(seasonal+pandemic), sym,med,nonflu | *total,unique,HCF*,RSV | Y | 8558 | 97.42 |
| 26 | flu terms(seasonal+pandemic), sym,med,authority | *total,unique,HCF*,RSV | N | 8316 | 130.9 |
| 27 | flu terms(seasonal+pandemic), sym,preventions | *total,unique,HCF*,RSV | N | 8149 | 114.2 |
| 28 | sym,med,nonflu,RSV | *total,unique,HCF*,flu terms(seasonal+pandemic) | N | 9091 | 70.62 |
| 29 | sym,med,nonflu,authority | *total,HCF*,RSV,flu terms(seasonal+pandemic) | N | 8304 | 102.1 |
| 30 | flu terms(seasonal+pandemic),sym, med,nonflu | *total,unique,HCF*,RSV,authority | N | 8247 | 79.68 |
| 31 | flu terms(seasonal+pandemic),sym,med,nonflu | *total,unique,HCF*,RSV,authority | N | 8157 | 90.93 |
| 32 | seasonal flu terms,sym,med,nonflu | *total,HCF*,RSV,pandemic flu terms,authority | N | 8239 | 89.66 |
| 33 | seasonal flu terms*,sym,med,nonflu* | *total,HCF*,RSV,pandemic flu terms,authority | N | 8455 | 85.58 |
| 34 | seasonal flu terms*,sym,med,nonflu* | *total,unique,HCF*,RSV,pandemic flu terms,authority | N | 7689 | 91.46 |
| 35 | seasonal flu terms*,pandemic flu terms,sym,med,nonflu*,authority | *total,uniqe,HCF*,RSV | N | 7622 | 164.4 |
| 36 | seasonal flu terms*,sym,med,nonflu | *total,unique,HCF,*RSV,pandemic flu terms,authority | N | 7538 | 95.38 |

Note:

In regular font: Google search index and RSV( laboratory surveillance data)

In italic font: HealthMap data

*: Common cold terms in English and Chinese are moved from flu terms to non-flu terms

## 2.4 Time lag effect

We also examined time lag effect for each surveillance system. We compared model performance with time lag varying from -2 weeks to +2 weeks for both pandemic and non-pandemic model. Model performance significantly improved with +1 week time lag for all the surveillance data series during the pandemic period, while no significant improvement was observed during the non-pandemic period (Table S5 and Table S6).

For pandemic period, $\boldsymbol{k}_{\boldsymbol{n},\boldsymbol{t}}^{\boldsymbol{p}}$ is grouped under $\theta_{j,t}$ and $\varphi_{j,t}$, and the time lag is applied to the individual group as described in Table S5. +1wk refers to for $k_{i,t}^{p}$ at time *t*, the response variable is $Y_{i,t+1}$ at time *t*+1. DIC and *pD* values are compared and T8 time lag model is selected for P model due to its significantly improved DIC value.

Table S5 Description for the time lag P model

|  | $\boldsymbol{\theta}_{\boldsymbol{j,t}}$ | $\boldsymbol{\varphi}_{\boldsymbol{j,t}}$ | DIC | *pD* |
| --- | --- | --- | --- | --- |
| T0 | +0wk | +0wk | 15660 | 1108 |
| T1 | +0wk | -1wk | 16860 | 1298 |
| T2 | +0wk | +1wk | 14240 | 947.8 |
| T3 | -1wk | -1wk | 19750 | 3970 |
| T4 | -1wk | +0wk | 22550 | 2608 |
| T5 | -1wk | +1wk | 19280 | 1322 |
| T6 | +1wk | -1wk | 13040 | 441 |
| T7 | +1wk | +0wk | 20990 | 902.1 |
| T8 | +1wk | +1wk | 9451 | 600.2 |

In NP model, for each informational environment index $k_{i,t}^{np}$ five sets of time-lag combination (-2wk, -1wk, 0wk, +1wk, +2wk) are included for comparison. Stepwise regression is conducted to provide guidance on selecting the top performing time lag models for further investigation. The combinations are then narrowed down to four as described in Table S6. DIC and *pD* are further assessed for T1, T2, T3 and T4, when compared to T0 ― the original NP model without any time lag component.

Table S6 Description for the time lag NP model

|  | T1 | | | T2 | | | T3 | | | T4 | | |
| --- | --- | --- | --- | --- | --- | --- | --- | --- | --- | --- | --- | --- |
|  | $k_{1}^{np}$ | $k_{2}^{np}$ | $k_{3}^{np}$ | $k_{1}^{np}$ | $k_{2}^{np}$ | $k_{3}^{np}$ | $k_{1}^{np}$ | $k_{2}^{np}$ | $k_{3}^{np}$ | $k_{1}^{np}$ | $k_{2}^{np}$ | $k_{3}^{np}$ |
| $\boldsymbol{Y}_{\boldsymbol{1,t}}$ | +2wk | -1wk | 0wk | +2wk | -1wk | -2wk | +2wk | +2wk | 0wk | +2wk | +2wk | -2wk |
| $\boldsymbol{Y}_{\boldsymbol{2,t}}$ | +2wk | 0wk | -1wk | +2wk | 0wk | -2wk | +2wk | +2wk | -1wk | +2wk | +2wk | -2wk |
| $\boldsymbol{Y}_{\boldsymbol{3,t}}$ | -1wk | -1wk | -2wk | +2wk | -1wk | -2wk | -1wk | +2wk | -2wk | +2wk | +2wk | -2wk |
| $\boldsymbol{Y}_{\boldsymbol{4,t}}$ | -2wk | -1wk | -1wk | -2wk | -1wk | +2wk | +2wk | -1wk | -1wk | +2wk | -1wk | +2wk |
| $\boldsymbol{Y}_{\boldsymbol{5,t}}$ | -2wk | 0wk | +2wk | +2wk | 0wk | +2wk | -2wk | +2wk | +2wk | +2wk | +2wk | +2wk |
| $\boldsymbol{Y}_{\boldsymbol{6,t}}$ | -2wk | +2wk | -1wk | -2wk | -2wk | -1wk | -2wk | +2wk | +2wk | -2wk | -2wk | +2wk |
| $\boldsymbol{Y}_{\boldsymbol{7,t}}$ | +2wk | -2wk | +1wk | +2wk | -2wk | +2wk | +2wk | +1wk | +1wk | +2wk | +1wk | +2wk |
| $\boldsymbol{Y}_{\boldsymbol{8,t}}$ | +2wk | -1wk | 0wk | +2wk | -1wk | -1wk | +2wk | +1wk | 0wk | +2wk | +1wk | -1wk |
| $\boldsymbol{Y}_{\boldsymbol{9,t}}$ | -2wk | 0wk | -1wk | -2wk | 0wk | +2wk | +2wk | 0wk | -1wk | +2wk | 0wk | +2wk |
| $\boldsymbol{Y}_{\boldsymbol{10,t}}$ | -1wk | +1wk | -1wk | -1wk | +1wk | +2wk | +2wk | +1wk | -1wk | +2wk | +1wk | +2wk |
| $\boldsymbol{Y}_{\boldsymbol{11,t}}$ | +2wk | -2wk | -2wk | +2wk | -2wk | +2wk | +2wk | +1wk | -2wk | +2wk | +1wk | +2wk |

Four out of the eleven data streams show lower DIC and *pD* values in the time lag models, among which, only the laboratory surveillance lab(% pos) shows a relative ratio of over 10% (Table S7). The difference among T1, T3, T4 and T2 is less than 3%, which suggests there is no substantial difference in model performance among the four time lag combinations.

Table S7 Model performance comparison among different time lag models for lab(%pos)

| Time Lag Model | Dbar | Dhat | DIC | pD | Relative Ratio |
| --- | --- | --- | --- | --- | --- |
| T1 | 58.17 | 49.1 | 67.24 | 9.074 | 0 |
| T3 | 58.61 | 49.53 | 67.69 | 9.079 | 0.0067 |
| T4 | 59.53 | 50.45 | 68.61 | 9.078 | 0.0204 |
| T2 | 60.06 | 50.98 | 69.14 | 9.076 | 0.0283 |
| T0 | 85.01 | 75.92 | 94.09 | 9.081 | 0.3993 |

## 2.5 Sensitivity analysis

To examine the sensitivity of the model to the choice of *prior*, models are run with three sets of hyper-parameters, as listed in Table S7.

Table S7 Non-informative priors for the sensitivity analysis

| Coefficient | *Prior* I | *Prior* II | *Prior* III |
| --- | --- | --- | --- |
| $\boldsymbol{\beta}_{\boldsymbol{j,t,m}}$ | dnorm(0,.01) | dnorm(0,.0001) | dnorm(0,1) |
| $\boldsymbol{\alpha}_{\boldsymbol{j,t,n}}$ | dnorm(0,.01) | dnorm(0,.0001) | dnorm(0,1) |
| $\boldsymbol{\rho}_{\boldsymbol{s,j,t,i}}$ | dnorm(0,.01) | dnorm(0,.0001) | dnorm(0,1) |
| $\boldsymbol{\tau}_{\boldsymbol{j}}$ | dgamma(.01,.01) | dgamma(.0001,.0001) | dgamma(1,1) |

# To explore the possibility of using informed prior distributions, in selected data streams in the NP model, the mean of the posterior distribution is used as the mean for the prior, while variance of 1 is used to give the model more flexibility.

# 3 Results

3.1 Pandemic model

Table S8 Posterior distributions for correspondence coefficient in P model

| **Coefficient** | **Surveillance System** | **Mean** | **SD** | **Lower 95%** | **Median** | **Upper 95%** |
| --- | --- | --- | --- | --- | --- | --- |
| **α1** | flu-HA | 4.8710 | 0.0363 | 4.8000 | 4.8710 | 4.9420 |
| **α2** | flu-HA | 0.3164 | 0.0243 | 0.2687 | 0.3164 | 0.3640 |
| **α3** | flu-HA | -0.0451 | 0.0255 | -0.0951 | -0.0450 | 0.0048 |
| **α4** | flu-HA | -0.2930 | 0.0220 | -0.3367 | -0.2928 | -0.2504 |
| **α5** | flu-HA | -0.2432 | 0.0193 | -0.2812 | -0.2432 | -0.2054 |
| **α6** | flu-HA | -0.2081 | 0.0092 | -0.2261 | -0.2081 | -0.1901 |
| **α7** | flu-HA | 0.3542 | 0.0164 | 0.3221 | 0.3542 | 0.3865 |
| **α1** | DFC | 7.1500 | 0.0103 | 7.1300 | 7.1500 | 7.1700 |
| **α2** | DFC | 0.2653 | 0.0075 | 0.2506 | 0.2653 | 0.2800 |
| **α3** | DFC | -0.1281 | 0.0077 | -0.1432 | -0.1281 | -0.1131 |
| **α4** | DFC | -0.2142 | 0.0061 | -0.2263 | -0.2142 | -0.2023 |
| **α5** | DFC | -0.1951 | 0.0059 | -0.2066 | -0.1951 | -0.1836 |
| **α6** | DFC | -0.1530 | 0.0027 | -0.1583 | -0.1530 | -0.1477 |
| **α7** | DFC | 0.2638 | 0.0049 | 0.2541 | 0.2638 | 0.2735 |
| **α1** | NID | 5.7260 | 0.0195 | 5.6880 | 5.7260 | 5.7640 |
| **α2** | NID | 0.2261 | 0.0127 | 0.2014 | 0.2261 | 0.2510 |
| **α3** | NID | 0.0487 | 0.0140 | 0.0212 | 0.0487 | 0.0761 |
| **α4** | NID | -0.4031 | 0.0134 | -0.4296 | -0.4030 | -0.3770 |
| **α5** | NID | -0.2153 | 0.0103 | -0.2356 | -0.2153 | -0.1951 |
| **α6** | NID | -0.2404 | 0.0052 | -0.2508 | -0.2403 | -0.2301 |
| **α7** | NID | 0.3850 | 0.0095 | 0.3664 | 0.3849 | 0.4036 |
| **α1** | GP | 1.6070 | 0.0360 | 1.5350 | 1.6070 | 1.6780 |
| **α2** | GP | 0.0087 | 0.0281 | -0.0472 | 0.0087 | 0.0647 |
| **α3** | GP | -0.0079 | 0.0294 | -0.0663 | -0.0079 | 0.0506 |
| **α4** | GP | -0.0178 | 0.0193 | -0.0560 | -0.0179 | 0.0205 |
| **α5** | GP | 0.0201 | 0.0218 | -0.0233 | 0.0201 | 0.0635 |
| **α6** | GP | -0.0007 | 0.0059 | -0.0124 | -0.0007 | 0.0110 |
| **α7** | GP | -0.0002 | 0.0140 | -0.0280 | -0.0002 | 0.0276 |
| **α1** | RHE | 0.1535 | 0.0303 | 0.0936 | 0.1534 | 0.2145 |
| **α2** | RHE | -0.0069 | 0.0279 | -0.0627 | -0.0068 | 0.0487 |
| **α3** | RHE | 0.0254 | 0.0221 | -0.0182 | 0.0255 | 0.0694 |
| **α4** | RHE | -0.0332 | 0.0179 | -0.0689 | -0.0333 | 0.0023 |
| **α5** | RHE | 0.0178 | 0.0240 | -0.0300 | 0.0178 | 0.0655 |
| **α6** | RHE | 0.0020 | 0.0054 | -0.0088 | 0.0020 | 0.0128 |
| **α7** | RHE | -0.0099 | 0.0125 | -0.0348 | -0.0099 | 0.0151 |
| **α1** | Lab(%positive) | 2.1630 | 0.0897 | 1.9850 | 2.1630 | 2.3420 |
| **α2** | Lab(%positive) | 0.1279 | 0.0700 | -0.0118 | 0.1278 | 0.2677 |
| **α3** | Lab(%positive) | -0.1669 | 0.0734 | -0.3131 | -0.1670 | -0.0207 |
| **α4** | Lab(%positive) | -0.0594 | 0.0481 | -0.1559 | -0.0592 | 0.0364 |
| **α5** | Lab(%positive) | -0.0698 | 0.0546 | -0.1786 | -0.0698 | 0.0393 |
| **α6** | Lab(%positive) | -0.0250 | 0.0148 | -0.0545 | -0.0250 | 0.0045 |
| **α7** | Lab(%positive) | 0.0057 | 0.0348 | -0.0637 | 0.0057 | 0.0751 |
| **α1** | P&I-HA | 6.8650 | 0.0220 | 6.8210 | 6.8650 | 6.9080 |
| **α2** | P&I-HA | -0.0494 | 0.0109 | -0.0707 | -0.0494 | -0.0281 |
| **α3** | P&I-HA | 0.1002 | 0.0115 | 0.0776 | 0.1002 | 0.1227 |
| **α4** | P&I-HA | -0.0059 | 0.0094 | -0.0243 | -0.0059 | 0.0125 |
| **α5** | P&I-HA | 0.0658 | 0.0117 | 0.0428 | 0.0658 | 0.0887 |
| **α6** | P&I-HA | -0.0017 | 0.0021 | -0.0059 | -0.0017 | 0.0024 |
| **α7** | P&I-HA | 0.0524 | 0.0094 | 0.0340 | 0.0524 | 0.0707 |
| **α1** | P&I-HA(0-15) | 5.0180 | 0.0512 | 4.9170 | 5.0180 | 5.1170 |
| **α2** | P&I-HA(0-15) | -0.0465 | 0.0234 | -0.0925 | -0.0465 | -0.0007 |
| **α3** | P&I-HA(0-15) | 0.2401 | 0.0258 | 0.1893 | 0.2402 | 0.2905 |
| **α4** | P&I-HA(0-15) | -0.0440 | 0.0226 | -0.0885 | -0.0439 | 0.0000 |
| **α5** | P&I-HA(0-15) | 0.2001 | 0.0253 | 0.1505 | 0.2000 | 0.2497 |
| **α6** | P&I-HA(0-15) | 0.0039 | 0.0047 | -0.0053 | 0.0039 | 0.0132 |
| **α7** | P&I-HA(0-15) | 0.0655 | 0.0215 | 0.0233 | 0.0656 | 0.1076 |
| **α1** | P&I-HA(65+) | 6.3590 | 0.0288 | 6.3020 | 6.3590 | 6.4160 |
| **α2** | P&I-HA(65+) | -0.0621 | 0.0144 | -0.0904 | -0.0621 | -0.0339 |
| **α3** | P&I-HA(65+) | 0.0458 | 0.0149 | 0.0165 | 0.0458 | 0.0749 |
| **α4** | P&I-HA(65+) | 0.0171 | 0.0119 | -0.0062 | 0.0171 | 0.0404 |
| **α5** | P&I-HA(65+) | -0.0170 | 0.0155 | -0.0474 | -0.0170 | 0.0132 |
| **α6** | P&I-HA(65+) | -0.0098 | 0.0028 | -0.0152 | -0.0098 | -0.0043 |
| **α7** | P&I-HA(65+) | 0.0335 | 0.0120 | 0.0100 | 0.0335 | 0.0570 |
| **α1** | Lab(#specimen) | 7.7930 | 0.0093 | 7.7750 | 7.7930 | 7.8110 |
| **α2** | Lab(#specimen) | 0.2129 | 0.0065 | 0.2002 | 0.2129 | 0.2256 |
| **α3** | Lab(#specimen) | 0.0054 | 0.0066 | -0.0075 | 0.0054 | 0.0182 |
| **α4** | Lab(#specimen) | -0.1938 | 0.0050 | -0.2036 | -0.1938 | -0.1839 |
| **α5** | Lab(#specimen) | -0.1794 | 0.0051 | -0.1894 | -0.1794 | -0.1694 |
| **α6** | Lab(#specimen) | -0.1171 | 0.0022 | -0.1215 | -0.1171 | -0.1128 |
| **α7** | Lab(#specimen) | 0.2060 | 0.0041 | 0.1980 | 0.2060 | 0.2140 |
| **α1** | Lab(#positive) | 6.1560 | 0.0183 | 6.1200 | 6.1560 | 6.1920 |
| **α2** | Lab(#positive) | 0.4211 | 0.0114 | 0.3988 | 0.4211 | 0.4435 |
| **α3** | Lab(#positive) | -0.0426 | 0.0126 | -0.0673 | -0.0426 | -0.0179 |
| **α4** | Lab(#positive) | -0.4336 | 0.0120 | -0.4573 | -0.4335 | -0.4102 |
| **α5** | Lab(#positive) | -0.2647 | 0.0092 | -0.2827 | -0.2647 | -0.2468 |
| **α6** | Lab(#positive) | -0.2457 | 0.0047 | -0.2548 | -0.2457 | -0.2365 |
| **α7** | Lab(#positive) | 0.3456 | 0.0085 | 0.3288 | 0.3456 | 0.3623 |
| **β1** | flu-HA | 0.0118 | 0.0006 | 0.0107 | 0.0118 | 0.0129 |
| **β2** | flu-HA | 0.0037 | 0.0004 | 0.0030 | 0.0037 | 0.0044 |
| **β3** | flu-HA | 0.0016 | 0.0001 | 0.0013 | 0.0016 | 0.0018 |
| **β4** | flu-HA | -0.0070 | 0.0006 | -0.0081 | -0.0070 | -0.0059 |
| **β5** | flu-HA | 0.0034 | 0.0003 | 0.0028 | 0.0034 | 0.0039 |
| **β1** | DFC | 0.0139 | 0.0002 | 0.0136 | 0.0139 | 0.0142 |
| **β2** | DFC | 0.0035 | 0.0001 | 0.0033 | 0.0035 | 0.0037 |
| **β3** | DFC | 0.0013 | 0.0000 | 0.0012 | 0.0013 | 0.0013 |
| **β4** | DFC | -0.0067 | 0.0002 | -0.0071 | -0.0067 | -0.0064 |
| **β5** | DFC | 0.0026 | 0.0001 | 0.0024 | 0.0026 | 0.0028 |
| **β1** | NID | 0.0189 | 0.0003 | 0.0183 | 0.0189 | 0.0194 |
| **β2** | NID | 0.0047 | 0.0002 | 0.0044 | 0.0047 | 0.0051 |
| **β3** | NID | 0.0018 | 0.0001 | 0.0017 | 0.0018 | 0.0019 |
| **β4** | NID | -0.0087 | 0.0003 | -0.0093 | -0.0087 | -0.0081 |
| **β5** | NID | 0.0037 | 0.0002 | 0.0034 | 0.0037 | 0.0040 |
| **β1** | GP | 0.0028 | 0.0006 | 0.0016 | 0.0028 | 0.0040 |
| **β2** | GP | 0.0004 | 0.0003 | -0.0003 | 0.0004 | 0.0011 |
| **β3** | GP | 0.0001 | 0.0001 | -0.0001 | 0.0001 | 0.0003 |
| **β4** | GP | -0.0004 | 0.0006 | -0.0017 | -0.0004 | 0.0008 |
| **β5** | GP | -0.0001 | 0.0003 | -0.0007 | -0.0001 | 0.0004 |
| **β1** | RHE | -0.0011 | 0.0005 | -0.0020 | -0.0011 | -0.0001 |
| **β2** | RHE | -0.0005 | 0.0004 | -0.0012 | -0.0005 | 0.0003 |
| **β3** | RHE | 0.0001 | 0.0001 | -0.0001 | 0.0001 | 0.0003 |
| **β4** | RHE | 0.0008 | 0.0005 | -0.0002 | 0.0008 | 0.0018 |
| **β5** | RHE | -0.0003 | 0.0002 | -0.0008 | -0.0003 | 0.0001 |
| **β1** | Lab(%positive) | 0.0051 | 0.0015 | 0.0022 | 0.0051 | 0.0080 |
| **β2** | Lab(%positive) | 0.0022 | 0.0009 | 0.0005 | 0.0022 | 0.0039 |
| **β3** | Lab(%positive) | 0.0006 | 0.0002 | 0.0002 | 0.0006 | 0.0010 |
| **β4** | Lab(%positive) | -0.0033 | 0.0016 | -0.0065 | -0.0033 | 0.0000 |
| **β5** | Lab(%positive) | -0.0012 | 0.0007 | -0.0026 | -0.0012 | 0.0003 |
| **β1** | P&I-HA | 0.0014 | 0.0003 | 0.0007 | 0.0014 | 0.0020 |
| **β2** | P&I-HA | 0.0005 | 0.0002 | 0.0001 | 0.0005 | 0.0008 |
| **β3** | P&I-HA | 0.0000 | 0.0000 | -0.0001 | 0.0000 | 0.0001 |
| **β4** | P&I-HA | -0.0001 | 0.0002 | -0.0005 | -0.0001 | 0.0004 |
| **β5** | P&I-HA | -0.0002 | 0.0002 | -0.0005 | -0.0002 | 0.0001 |
| **β1** | P&I-HA(0-15) | 0.0049 | 0.0008 | 0.0034 | 0.0049 | 0.0064 |
| **β2** | P&I-HA(0-15) | 0.0017 | 0.0004 | 0.0010 | 0.0017 | 0.0025 |
| **β3** | P&I-HA(0-15) | -0.0004 | 0.0001 | -0.0006 | -0.0004 | -0.0002 |
| **β4** | P&I-HA(0-15) | -0.0008 | 0.0005 | -0.0018 | -0.0008 | 0.0002 |
| **β5** | P&I-HA(0-15) | 0.0004 | 0.0004 | -0.0003 | 0.0004 | 0.0011 |
| **β1** | P&I-HA(65+) | 0.0011 | 0.0005 | 0.0002 | 0.0011 | 0.0020 |
| **β2** | P&I-HA(65+) | -0.0004 | 0.0002 | -0.0008 | -0.0004 | 0.0001 |
| **β3** | P&I-HA(65+) | 0.0002 | 0.0001 | 0.0001 | 0.0002 | 0.0003 |
| **β4** | P&I-HA(65+) | 0.0001 | 0.0003 | -0.0005 | 0.0001 | 0.0007 |
| **β5** | P&I-HA(65+) | -0.0002 | 0.0002 | -0.0006 | -0.0002 | 0.0002 |
| **β1** | Lab(#specimen) | 0.0076 | 0.0002 | 0.0073 | 0.0076 | 0.0079 |
| **β2** | Lab(#specimen) | 0.0016 | 0.0001 | 0.0014 | 0.0016 | 0.0017 |
| **β3** | Lab(#specimen) | 0.0013 | 0.0000 | 0.0012 | 0.0013 | 0.0013 |
| **β4** | Lab(#specimen) | -0.0062 | 0.0002 | -0.0065 | -0.0062 | -0.0059 |
| **β5** | Lab(#specimen) | 0.0029 | 0.0001 | 0.0027 | 0.0029 | 0.0030 |
| **β1** | Lab(#positive) | 0.0147 | 0.0003 | 0.0142 | 0.0147 | 0.0153 |
| **β2** | Lab(#positive) | 0.0047 | 0.0002 | 0.0043 | 0.0047 | 0.0050 |
| **β3** | Lab(#positive) | 0.0021 | 0.0001 | 0.0020 | 0.0021 | 0.0022 |
| **β4** | Lab(#positive) | -0.0096 | 0.0003 | -0.0102 | -0.0096 | -0.0091 |
| **β5** | Lab(#positive) | 0.0046 | 0.0001 | 0.0043 | 0.0046 | 0.0048 |
| **τ** | GP | 310.1 | 138 | 101.2 | 290 | 633.7 |
| **τ** | RHE | 346.7 | 155.5 | 111.6 | 323.7 | 712.9 |
| **τ** | Lab(%positive) | 50.04 | 22.36 | 16.27 | 46.77 | 102.5 |

Table S9 Model performance comparison between models fed with incidence rate of all-age group versus 5-14yr

|  | Incidence rate (5-14yr) | | Incidence rate (all-age) | | |
| --- | --- | --- | --- | --- | --- |
|  | DIC | pD | DIC | pD |  |
| flu-HA | 784.1 | 12.68 | 809.5 | 12.01 |  |
| DFC | 3250 | 19.83 | 3703 | 12.18 |  |
| NID | 2332 | 16.12 | 2717 | 12.15 |  |
| GP | -64.43 | 14.22 | -53.64 | 14.2 |  |
| RHE | -27.47 | 14.2 | -29.82 | 14.19 |  |
| Lab(%pos) | 16.71 | 14.12 | 12.68 | 14.16 |  |
| P&I-HA | 352.6 | 14.24 | 349.3 | 12.07 |  |
| P&I-HA(0-15) | 299.1 | 12.56 | 315.4 | 12.03 |  |
| P&I-HA(65+) | 240 | 13.92 | 234.8 | 12.03 |  |
| Lab(#spm) | 4817 | 20.57 | 4195 | 12.22 |  |
| Lab(#pos) | 4116 | 15.67 | 3992 | 12.18 |  |

3.2 Non-pandemic model

Table S10 Posterior distributions for correspondence coefficient in NP model

|  |  | **Flu Season** | | **Non-flu season** | |  |
| --- | --- | --- | --- | --- | --- | --- |
| **Coefficient** | Y | Mean | 95% CI | Mean | 95% CI |  |
| **ρ1** | flu-HA | 1.973 | (1.901, 2.045) | 1.243 | (1.174, 1.312) |  |
| **ρ2** | flu-HA | -0.058 | (-0.185, 0.070) | -0.219 | (-0.355, -0.083) |  |
| **ρ3** | flu-HA | -0.120 | (-0.272, 0.031) | -0.003 | (-0.249, 0.244) |  |
| **ρ4** | flu-HA | 0.447 | (0.221, 0.673) | 0.219 | (0.030, 0.407) |  |
| **ρ1** | P&I-HA | 2.991 | (2.978, 3.004) | 2.889 | (2.887, 2.901) |  |
| **ρ2** | P&I-HA | -0.012 | (-0.034, 0.011) | -0.003 | (-0.027, 0.021) |  |
| **ρ3** | P&I-HA | 0.008 | (-0.019, 0.035) | -0.006 | (-0.050, 0.037) |  |
| **ρ4** | P&I-HA | 0.071 | (0.031, 0.111) | 0.032 | (-0.001, 0.066) |  |
| **ρ1** | GOPC | 0.753 | (0.728, 0.777) | 0.559 | (0.535, 0.582) |  |
| **ρ2** | GOPC | 0.005 | (-0.038, 0.049) | -0.036 | (-0.083, 0.010) |  |
| **ρ3** | GOPC | -0.017 | (-0.068, 0.035) | 0.029 | (-0.054, 0.114) |  |
| **ρ4** | GOPC | 0.119 | (0.041, 0.196) | -0.056 | (-0.121, 0.008) |  |
| **ρ1** | GP | 1.691 | (1.674, 1.708) | 1.610 | (1.594,1.626) |  |
| **ρ2** | GP | -0.029 | (-0.059, 0.001) | -0.046 | (-0.078, -0.014) |  |
| **ρ3** | GP | -0.014 | (-0.050, 0.022) | 0.003 | (-0.054, 0.061) |  |
| **ρ4** | GP | 0.077 | (0.024, 0.130) | -0.034 | (-0.078, 0.011) |  |
| **ρ1** | RHE | 0.175 | (0.157, 0.193) | 0.124 | (0.106, 0.141) |  |
| **ρ2** | RHE | -0.026 | (-0.058, 0.006) | 0.025 | (-0.009, 0.060) |  |
| **ρ3** | RHE | 0.025 | (-0.013, 0.064) | -0.003 | (-0.065, 0.059) |  |
| **ρ4** | RHE | 0.055 | (-0.002, 0.112) | -0.008 | (-0.055, 0.040) |  |
| **ρ1** | Lab(%positive) | -0.724 | (-0.794, -0.654) | -1.342 | (-1.409, -1.275) |  |
| **ρ2** | Lab(%positive) | 0.001 | (-0.124, 0.126) | -0.236 | (-0.370, -0.103) |  |
| **ρ3** | Lab(%positive) | -0.094 | (-0.243, 0.054) | -0.037 | (-0.278, 0.203) |  |
| **ρ4** | Lab(%positive) | 0.304 | (0.082, 0.526) | 0.196 | (0.011, 0.381) |  |
| **ρ1** | CCC/KG | -1.008 | (-1.038, -0.978) | -1.140 | (-1.168, -1.112) |  |
| **ρ2** | CCC/KG | -0.007 | (-0.059, 0.046) | 0.004 | (-0.052, 0.060) |  |
| **ρ3** | CCC/KG | -0.016 | (-0.078, 0.047) | -0.084 | (-0.186, 0.018) |  |
| **ρ4** | CCC/KG | 0.142 | (0.048, 0.235) | -0.056 | (-0.134, 0.022) |  |
| **ρ1** | P&I-HA(0-15) | 2.111 | (2.084, 2.138) | 1.909 | (1.883, 1.935) |  |
| **ρ2** | P&I-HA(0-15) | -0.039 | (-0.087, 0.009) | -0.044 | (-0.095, 0.007) |  |
| **ρ3** | P&I-HA(0-15) | -0.064 | (-0.120, -0.007) | -0.004 | (-0.096, 0.089) |  |
| **ρ4** | P&I-HA(0-15) | 0.317 | (0.232, 0.402) | 0.075 | (0.004, 0.146) |  |
| **ρ1** | P&I-HA(65+) | 2.858 | (2.846, 2.870) | 2.775 | (2.764, 2.786) |  |
| **ρ2** | P&I-HA(65+) | -0.009 | (-0.030, 0.012) | 0.006 | (-0.017, 0.029) |  |
| **ρ3** | P&I-HA(65+) | 0.022 | (-0.003, 0.048) | -0.011 | (-0.052, 0.031) |  |
| **ρ4** | P&I-HA(65+) | 0.029 | (-0.009, 0.067) | 0.025 | (-0.006, 0.057) |  |
| **ρ1** | Lab(#specimen) | 2.972 | (2.946, 2.998) | 2.869 | (2.844, 2.894) |  |
| **ρ2** | Lab(#specimen) | -0.020 | (-0.066, 0.026) | 0.011 | (-0.037, 0.060) |  |
| **ρ3** | Lab(#specimen) | -0.012 | (-0.067, 0.042) | -0.020 | (-0.109, 0.068) |  |
| **ρ4** | Lab(#specimen) | 0.131 | (0.050, 0.213) | 0.024 | (-0.044, 0.092) |  |
| **ρ1** | Lab(#positive) | 2.248 | (2.170, 2.326) | 1.527 | (1.452,1.602) |  |
| **ρ2** | Lab(#positive) | -0.019 | (-0.157, 0.120) | -0.225 | (-0.373, -0.077) |  |
| **ρ3** | Lab(#positive) | -0.107 | (-0.271, 0.058) | -0.058 | (-0.325, 0.210) |  |
| **ρ4** | Lab(#positive) | 0.435 | (0.189, 0.681) | 0.220 | (0.016, 0.425) |  |
| **τ** | flu-HA | 8.145 | (6.154, 10.41) |  |  |  |
| **τ** | P&I-HA | 258.900 | (195.5, 331.0) |  |  |  |
| **τ** | GOPC | 69.690 | (52.62, 89.08) |  |  |  |
| **τ** | GP | 146.400 | (110.6, 187.1) |  |  |  |
| **τ** | RHE | 126.800 | (95.76, 162) |  |  |  |
| **τ** | Lab(%positive) | 8.476 | (6.402,10.84) |  |  |  |
| **τ** | CCC | 47.610 | (35.97, 60.84) |  |  |  |
| **τ** | P&I-HA(0-15YR) | 57.730 | (43.6, 73.79) |  |  |  |
| **τ** | P&I-HA(65+YR) | 292.700 | (221.1, 374.4) |  |  |  |
| **τ** | Lab(#specimen) | 63.100 | (47.66, 80.69) |  |  |  |
| **τ** | Lab(#positive) | 6.894 | (5.207, 8.811) |  |  |  |

References

1. Centers for Disease Control and Prevention (CDC): **Fluview**. Retrieved 10/15, 2013, from [<http://www.cdc.gov/flu/weekly/>]
2. Spiegelhalter DJ, Best NG, Carlin BP, Van Der Linde A: **Bayesian measures of model complexity and fit.** *Journal of the Royal Statistical Society: Series B (Statistical Methodology)* 2002, **6**4(4):583-639.
3. **Zhang Y**. "From Noise to Characterization Tool: Assessing Biases and Performance of Influenza Surveillance Methods through Qualitative and Quantitative Studies." Dissertation, Georgetown University, 2013. Ann Arbor: ProQuest. Web. 28 Aug. 2013. (Publication No. AAT 3559767)
