# Supplementary material for: Characterizing Influenza surveillance systems performance: application of a Bayesian hierarchical statistical model to Hong Kong surveillance data
Source: BMC Public Health. 2014 Aug 15;14:850. doi: 10.1186/1471-2458-14-850 (PMC4246552; doi:10.1186/1471-2458-14-850)
Supplement: Supplementary file 2 — Additional file 2: OpenBUGS codes for the final pandemic and non-pandemic model. (DOCX 23 KB) [file 12889_2014_7289_MOESM2_ESM.docx]

Additional file II

# 1 Pandemic Model Codes

model

{

for( t in 1:N) {

xs[t]<-x[t]*10000

y1[t]~dpois(lambda1[t])

#comments: y1 for influenza hospitalization in count

y2[t]~dpois(lambda2[t])

#comments: y2 for ILI-related Designated Flu Clinics visits in count

y3[t]~dpois(lambda3[t])

#comments: y3 for cases reported to Notifiable Infectious Disease reporting system in count

y4[t]~dnorm(m4[t], tau.e[1])

#comments: y4 for ILI-related private physician visits in per 1,000 visit after log transformation

y5[t]~dnorm(m5[t], tau.e[2])

#comments: y5 for case of fever per 1,000 residents at elderly home after log transformation

y6[t]~dnorm(m6[t], tau.e[3])

#comments: y6 for influenza positive laboratory samples per 1,000 specimens received after log transformation

y7[t]~dpois(lambda5[t])

#comments: y7 for P&I hospitalization for all age in count

y8[t]~dpois(lambda6[t])

#comments: y8 for P&I hospitalization for 0-15yr in count

y9[t]~dpois(lambda7[t])

#comments: y9 for P&I hospitalization for 65+yr in count

y12[t]~dpois(lambda12[t])

#comments: y12 for the number of specimen received in count

y13[t]~dpois(lambda13[t])

#comments: y13 for the number of sample tested positive for influenza virus

log(lambda1[t])<-xs[t]*theta[1,t]+phi[1,t]

log(lambda2[t])<-xs[t]*theta[2,t]+phi[2,t]

log(lambda3[t])<-xs[t]*theta[3,t]+phi[3,t]

m4[t]<-xs[t]*theta[4,t]+phi[4,t]

m5[t]<-xs[t]*theta[5,t]+phi[5,t]

m6[t]<-xs[t]*theta[6,t]+phi[6,t]

log(lambda5[t])<-xs[t]*theta[7,t]+phi[7,t]

log(lambda6[t])<-xs[t]*theta[8,t]+phi[8,t]

log(lambda7[t])<-xs[t]*theta[9,t]+phi[9,t]

log(lambda12[t])<-xs[t]*theta[10,t]+phi[10,t]

log(lambda13[t])<-xs[t]*theta[11,t]+phi[11,t]

#x[t] is the percentage of new cases among the total population

for ( i in 1:11) {

theta[i,t]<-beta[i,1]+beta[i,2]*k1[t]+beta[i,3]*k2[t]+beta[i,4]*k3[t]+beta[i,5]*k4[t]

phi[i,t]<-alpha[i,1]+alpha[i,2]*k5[t]+alpha[i,3]*k6[t]+alpha[i,4]*k7[t]+alpha[i,5]*k8[t]+alpha[i,6]*k9[t]+alpha[i,7]*k10[t]

#comments: k1:search index for seasonal flu terms(except common cold), K2: symptoms, k3: medications, k4: nonflu(including common cold), k5: healthmap total news count; k6: healthmap unique news count; k7: healthcare facilities; k8: RSV; k9: search index for authority and pandemic; k10:search index for pandemic flu term.

}

}

for ( i in 1:11) {

for ( m in 1:5) {

beta[i,m] ~ dnorm(0, 0.01)}

for ( l in 1:7) {

alpha[i,l] ~ dnorm(0, 0.01)}

}

for (k in 1:3){

tau.e[k] ~ dgamma(.01,.01)}

}

Initial values:

1^st^ set:

list(alpha=structure(.Data=c(0.005,0.005,0.005,0.005,0.005,0.005,0.005,0.005,0.005,0.005,0.005,0.005,0.005,0.005,0.005,0.005,0.005,0.005,0.005,0.005,0.005,0.005,0.005,0.005,0.005,0.005,0.005,0.005,0.005,0.005,0.005,0.005,0.005,0.005,0.005,0.005,0.005,0.005,0.005,0.005,0.005,0.005,0.005,0.005,0.005,0.005,0.005,0.005,0.005,0.005,0.005,0.005,0.005,0.005,0.005,0.005,0.005,0.005,0.005,0.005,0.005,0.005,0.005,0.005,0.005,0.005,0.005,0.005,0.005,0.005,0.005,0.005,0.005,0.005,0.005,0.005,0.005), .Dim=c(11,7)), beta=structure(.Data=c(0.005,0.005,0.005,0.005,0.005,0.005,0.005,0.005,0.005,0.005,0.005,0.005,0.005,0.005,0.005,0.005,0.005,0.005,0.005,0.005,0.005,0.005,0.005,0.005,0.005,0.005,0.005,0.005,0.005,0.005,0.005,0.005,0.005,0.005,0.005,0.005,0.005,0.005,0.005,0.005,0.005,0.005,0.005,0.005,0.005,0.005,0.005,0.005,0.005,0.005,0.005,0.005,0.005,0.005,0.005), .Dim=c(11,5)),tau.e=c(0.05,0.05,0.005))

2^nd^ set:

list(alpha=structure(.Data=c(-0.01,-0.01,-0.01,-0.01,-0.01,-0.01,-0.01,-0.01,-0.01,-0.01,-0.01,-0.01,-0.01,-0.01,-0.01,-0.01,-0.01,-0.01,-0.01,-0.01,-0.01,-0.01,-0.01,-0.01,-0.01,-0.01,-0.01,-0.01,-0.01,-0.01,-0.01,-0.01,-0.01,-0.01,-0.01,-0.01,-0.01,-0.01,-0.01,-0.01,-0.01,-0.01,-0.01,-0.01,-0.01,-0.01,-0.01,-0.01,-0.01,-0.01,-0.01,-0.01,-0.01,-0.01,-0.01,-0.01,-0.01,-0.01,-0.01,-0.01,-0.01,-0.01,-0.01,-0.01,-0.01,-0.01,-0.01,-0.01,-0.01,-0.01,-0.01,-0.01,-0.01,-0.01,-0.01,-0.01,-0.01), .Dim=c(11,7)), beta=structure(.Data=c(-0.01,-0.01,-0.01,-0.01,-0.01,-0.01,-0.01,-0.01,-0.01,-0.01,-0.01,-0.01,-0.01,-0.01,-0.01,-0.01,-0.01,-0.01,-0.01,-0.01,-0.01,-0.01,-0.01,-0.01,-0.01,-0.01,-0.01,-0.01,-0.01,-0.01,-0.01,-0.01,-0.01,-0.01,-0.01,-0.01,-0.01,-0.01,-0.01,-0.01,-0.01,-0.01,-0.01,-0.01,-0.01,-0.01,-0.01,-0.01,-0.01,-0.01,-0.01,-0.01,-0.01,-0.01,-0.01), .Dim=c(11,5)),tau.e=c(0.01,0.01,0.01))

3^rd^ set:

list(alpha=structure(.Data=c(0.003,0.003,0.003,0.003,0.003,0.003,0.003,0.003,0.003,0.003,0.003,0.003,0.003,0.003,0.003,0.003,0.003,0.003,0.003,0.003,0.003,0.003,0.003,0.003,0.003,0.003,0.003,0.003,0.003,0.003,0.003,0.003,0.003,0.003,0.003,0.003,0.003,0.003,0.003,0.003,0.003,0.003,0.003,0.003,0.003,0.003,0.003,0.003,0.003,0.003,0.003,0.003,0.003,0.003,0.003,0.003,0.003,0.003,0.003,0.003,0.003,0.003,0.003,0.003,0.003,0.003,0.003,0.003,0.003,0.003,0.003,0.003,0.003,0.003,0.003,0.003,0.003), .Dim=c(11,7)), beta=structure(.Data=c(0.003,0.003,0.003,0.003,0.003,0.003,0.003,0.003,0.003,0.003,0.003,0.003,0.003,0.003,0.003,0.003,0.003,0.003,0.003,0.003,0.003,0.003,0.003,0.003,0.003,0.003,0.003,0.003,0.003,0.003,0.003,0.003,0.003,0.003,0.003,0.003,0.003,0.003,0.003,0.003,0.003,0.003,0.003,0.003,0.003,0.003,0.003,0.003,0.003,0.003,0.003,0.003,0.003,0.003,0.003), .Dim=c(11,5)),tau.e=c(0.03,0.03,0.003))

# 2 Non-pandemic Model Codes

model

{

for( t in 1:N) {

y1[t]~dnorm(m1[t], tau.e[1])

#comments: y1 for influenza hospitalization in count

y2[t]~dnorm(m2[t], tau.e[2])

#comments: y2 for P&I hospitalization in count

y3[t]~dnorm(m3[t], tau.e[3])

#comments: y3 for ILI cases at General Outpatient Department per 1,000 consultations after log transformation.

y4[t]~dnorm(m4[t], tau.e[4])

#comments: y4 for ILI-related private physician visits in per 1,000 visits after log transformation.

y5[t]~dnorm(m5[t], tau.e[5])

#comments: y5 for fever cases per 1,000 residents at elderly home after log transformation.

y6[t]~dnorm(m6[t], tau.e[6])

#comments: y6 for influenza positive laboratory samples per 1,000 specimens received after log transformation.

y7[t]~dnorm(m7[t], tau.e[7])

#comments: y7 for fever cases per 1,000 residents at daycare centre after log transformation.

y8[t]~dnorm(m8[t], tau.e[8])

#comments: y2 for 0-15 yr P&I hospitalization in count

y9[t]~dnorm(m9[t], tau.e[9])

#comments: y2 for 65+ yr P&I hospitalization in count

y10[t]~dnorm(m10[t], tau.e[10])

#comments: y10 for the number of specimen received at the laboratory

y11[t]~dnorm(m11[t], tau.e[11])

#comments: y11 for the number of specimen positive for influenza viruses

m1[t]<-u[1,t]

m2[t]<-u[2,t]

m3[t]<-u[3,t]

m4[t]<-u[4,t]

m5[t]<-u[5,t]

m6[t]<-u[6,t]

m7[t]<-u[7,t]

m8[t]<-u[8,t]

m9[t]<-u[9,t]

m10[t]<-u[10,t]

m11[t]<-u[11,t]

for ( j in 1:11){

u[j,t]<-(rho1[j,1]+rho1[j,2]*k1[t]+rho1[j,3]*k2[t]+rho1[j,4]*k3[t])*Ind[t]+(rho2[j,1]+rho2[j,2]*k1[t]+rho2[j,3]*k2[t]+rho2[j,4]*k3[t])*(1-Ind[t])

#K1: non-flu, k2: sickness index, k3: public awareness

#Ind: index for flu season versus non-flu season

}

}

for ( j in 1:11) {

for ( m in 1:4) {

rho1[j,m] ~ dnorm(0, 0.01)

rho2[j,m] ~ dnorm(0, 0.01)

}

}

for (k in 1:11){

tau.e[k] ~ dgamma(.01,.01)

}

}

Initial values:

1^st^ set:

list(rho11=structure(.Data=c(0.001,0.001,0.001,0.001,0.001,0.001,0.001,0.001,0.001,0.001,0.001,0.001,0.001,0.001,0.001,0.001,0.001,0.001,0.001,0.001,0.001,0.001,0.001,0.001,0.001,0.001,0.001,0.001,0.001,0.001,0.001,0.001,0.001,0.001,0.001,0.001,0.001,0.001,0.001,0.001,0.001,0.001,0.001,0.001), .Dim=c(11,4)),rho12=structure(.Data=c(0.001,0.001,0.001,0.001,0.001,0.001,0.001,0.001,0.001,0.001,0.001,0.001,0.001,0.001,0.001,0.001,0.001,0.001,0.001,0.001,0.001,0.001,0.001,0.001,0.001,0.001,0.001,0.001,0.001,0.001,0.001,0.001,0.001,0.001,0.001,0.001,0.001,0.001,0.001,0.001,0.001,0.001,0.001,0.001), .Dim=c(11,4)),rho21=structure(.Data=c(0.001,0.001,0.001,0.001,0.001,0.001,0.001,0.001,0.001,0.001,0.001,0.001,0.001,0.001,0.001,0.001,0.001,0.001,0.001,0.001,0.001,0.001,0.001,0.001,0.001,0.001,0.001,0.001,0.001,0.001,0.001,0.001,0.001,0.001,0.001,0.001,0.001,0.001,0.001,0.001,0.001,0.001,0.001,0.001), .Dim=c(11,4)),rho22=structure(.Data=c(0.001,0.001,0.001,0.001,0.001,0.001,0.001,0.001,0.001,0.001,0.001,0.001,0.001,0.001,0.001,0.001,0.001,0.001,0.001,0.001,0.001,0.001,0.001,0.001,0.001,0.001,0.001,0.001,0.001,0.001,0.001,0.001,0.001,0.001,0.001,0.001,0.001,0.001,0.001,0.001,0.001,0.001,0.001,0.001), .Dim=c(11,4)),tau.e=c(1,1,1,1,1,1,1,1,1,1,1))

2^nd^ set:

list(rho11=structure(.Data=c(0.05,0.05,0.05,0.05,0.05,0.05,0.05,0.05,0.05,0.05,0.05,0.05,0.05,0.05,0.05,0.05,0.05,0.05,0.05,0.05,0.05,0.05,0.05,0.05,0.05,0.05,0.05,0.05,0.05,0.05,0.05,0.05,0.05,0.05,0.05,0.05,0.05,0.05,0.05,0.05,0.05,0.05,0.05,0.05), .Dim=c(11,4)),rho12=structure(.Data=c(0.05,0.05,0.05,0.05,0.05,0.05,0.05,0.05,0.05,0.05,0.05,0.05,0.05,0.05,0.05,0.05,0.05,0.05,0.05,0.05,0.05,0.05,0.05,0.05,0.05,0.05,0.05,0.05,0.05,0.05,0.05,0.05,0.05,0.05,0.05,0.05,0.05,0.05,0.05,0.05,0.05,0.05,0.05,0.05), .Dim=c(11,4)),rho21=structure(.Data=c(0.05,0.05,0.05,0.05,0.05,0.05,0.05,0.05,0.05,0.05,0.05,0.05,0.05,0.05,0.05,0.05,0.05,0.05,0.05,0.05,0.05,0.05,0.05,0.05,0.05,0.05,0.05,0.05,0.05,0.05,0.05,0.05,0.05,0.05,0.05,0.05,0.05,0.05,0.05,0.05,0.05,0.05,0.05,0.05), .Dim=c(11,4)),rho22=structure(.Data=c(0.05,0.05,0.05,0.05,0.05,0.05,0.05,0.05,0.05,0.05,0.05,0.05,0.05,0.05,0.05,0.05,0.05,0.05,0.05,0.05,0.05,0.05,0.05,0.05,0.05,0.05,0.05,0.05,0.05,0.05,0.05,0.05,0.05,0.05,0.05,0.05,0.05,0.05,0.05,0.05,0.05,0.05,0.05,0.05), .Dim=c(11,4)),tau.e=c(0.05,0.05,0.05,0.05,0.05,0.05,0.05,0.05,0.05,0.05,0.05))

3^rd^ set:

list(rho11=structure(.Data=c(0.002,0.002,0.002,0.002,0.002,0.002,0.002,0.002,0.002,0.002,0.002,0.002,0.002,0.002,0.002,0.002,0.002,0.002,0.002,0.002,0.002,0.002,0.002,0.002,0.002,0.002,0.002,0.002,0.002,0.002,0.002,0.002,0.002,0.002,0.002,0.002,0.002,0.002,0.002,0.002,0.002,0.002,0.002,0.002), .Dim=c(11,4)),rho12=structure(.Data=c(0.002,0.002,0.002,0.002,0.002,0.002,0.002,0.002,0.002,0.002,0.002,0.002,0.002,0.002,0.002,0.002,0.002,0.002,0.002,0.002,0.002,0.002,0.002,0.002,0.002,0.002,0.002,0.002,0.002,0.002,0.002,0.002,0.002,0.002,0.002,0.002,0.002,0.002,0.002,0.002,0.002,0.002,0.002,0.002), .Dim=c(11,4)),rho21=structure(.Data=c(0.002,0.002,0.002,0.002,0.002,0.002,0.002,0.002,0.002,0.002,0.002,0.002,0.002,0.002,0.002,0.002,0.002,0.002,0.002,0.002,0.002,0.002,0.002,0.002,0.002,0.002,0.002,0.002,0.002,0.002,0.002,0.002,0.002,0.002,0.002,0.002,0.002,0.002,0.002,0.002,0.002,0.002,0.002,0.002), .Dim=c(11,4)),rho22=structure(.Data=c(0.002,0.002,0.002,0.002,0.002,0.002,0.002,0.002,0.002,0.002,0.002,0.002,0.002,0.002,0.002,0.002,0.002,0.002,0.002,0.002,0.002,0.002,0.002,0.002,0.002,0.002,0.002,0.002,0.002,0.002,0.002,0.002,0.002,0.002,0.002,0.002,0.002,0.002,0.002,0.002,0.002,0.002,0.002,0.002), .Dim=c(11,4)),tau.e=c(0.5,0.5,0.5,0.5,0.5,0.5,0.5,0.5,0.5,0.5,0.5))
